# Improved Method for Drawing of a Glycan Map, and the First Page of Glycan Atlas, Which Is a Compilation of Glycan Maps for a Whole Organism

**DOI:** 10.1371/journal.pone.0102219

**Published:** 2014-07-09

**Authors:** Shunji Natsuka, Mayumi Masuda, Wataru Sumiyoshi, Shin-ichi Nakakita

**Affiliations:** 1 Department of Biology, Faculty of Science, Niigata University, Niigata, Japan; 2 Life Science Research Center, Kagawa University, Takamatsu, Kagawa, Japan; Faculdade de Medicina, Universidade de São Paulo, Brazil

## Abstract

Glycan Atlas is a set of glycan maps over the whole body of an organism. The glycan map that includes data of glycan structure and quantity displays micro-heterogeneity of the glycans in a tissue, an organ, or cells. The two-dimensional glycan mapping is widely used for structure analysis of *N*-linked oligosaccharides on glycoproteins. In this study we developed a comprehensive method for the mapping of both *N*- and *O*-glycans with and without sialic acid. The mapping data of 150 standard pyridylaminated glycans were collected. The empirical additivity rule which was proposed in former reports was able to adapt for this extended glycan map. The adapted rule is that the elution time of pyridylamino glycans on high performance liquid chromatography (HPLC) is expected to be the simple sum of the partial elution times assigned to each monosaccharide residue. The comprehensive mapping method developed in this study is a powerful tool for describing the micro-heterogeneity of the glycans. Furthermore, we prepared 42 pyridylamino (PA-) glycans from human serum and were able to draw the map of human serum *N*- and *O*-glycans as an initial step of Glycan Atlas editing.

## Introduction

The two-dimensional mapping method for fluorescence-labeled glycans was first performed with high voltage-paper-electrophoresis [1]. Later, the high voltage paper electrophoresis was replaced with HPLC, resulting in dramatic improvement in the accuracy and reproducibility of the method [2], [3]. However, some limitations still remained. First, the analysis had to be performed separately for neutral and sialylated glycans [4]. Secondly, different HPLC conditions were used for the *O*-glycan and *N*-glycan mappings [5]. In this study we have solved those two problems. By using single glycan map, the present method allows the display of both *N*- and *O*-glycans irrespective of sialylation.

The elution time of a glycan on HPLC is influenced by slight differences in the experimental conditions, such as differences in the solvent composition, column deterioration, and temperature. Thus, in general, it is best to normalize the elution time using standard material(s) to obtain a high reproducibility of experiments. PA-isomaltooligosaccharides (IMO) has often been used as the standard material for size-fractionation and reversed phase HPLC of PA-glycans [6]. The elution time on size-fractionation HPLC is converted to an *S* value as glucose unit by normalization of PA-IMO. However, in the case of reversed phase HPLC, normalization by PA-IMO is not highly precise, because PA-glycans and PA-IMO are differentially influenced by the fluctuation of the experimental conditions. Therefore, a new scale for elution time on reversed phase HPLC, which was named the reversed phase scale, was developed to obtain high reliability [7]. The reversed phase scale presents the elution times as *R* values by using eight standard PA-glycans. In this study we determined the *R* and *S* values of 150 PA-glycans by using these new analytical conditions.

An empirical additivity rule concerning elution times on reversed phase HPLC was first deduced for high mannose-type glycans by Hase *et al.* [2]. Late, the rule was extended to neutral *N*-glycans [8], [9]. The rule is based on the observation that contribution to the elution time on reversed phase HPLC of a certain monosaccharide addition to a PA-glycan is scarcely influenced by other sugar residues. The degree of contribution is evaluated as a partial elution time that is specific to a position and kind of monosaccharides. In this study we determined partial elution times of *N*-glycans under new analytical condition and extended the rule to sialylated *N*-glycans.

Recently, the importance of three parameters for indicating glycan-distribution-pattern has become increasingly highlighted. These parameters can be abbreviated as the 3Ds: diversity, density, and depth of glycan distribution. The glycan mapping method presented in this paper is a powerful tool for displaying quantitative data of the first parameter, glycan diversity. Using this method, we have begun to develop Glycan Atlas, a set of glycan maps for the whole body of an organism. The data presented here are the base for making the glycan maps of organs and tissues.

## Materials and Methods

### Materials

The PA-glycans were purchased from Takara Bio Inc. (Kyoto, Japan), Seikagaku Corporation (Tokyo, Japan) or Masuda Chemical Industries Co., Ltd. (Takamatsu, Japan). The PA-glycans which were not commercially available were prepared as described elsewhere [5], [7]. Reagents were purchased from Wako Pure Chemical Industries, Inc. (Osaka, Japan) or Nakalai Tesque, Inc. (Kyoto, Japan). *Arthrobacter* sialidase was purchased from Nakalai Tesque, Inc. and α2-3sialidase was from Takara Bio Inc.

### High performance liquid chromatography

HPLC was performed using a Waters Alliance HPLC system with a Waters 2475 fluorescence spectrophotometer (Milford, MA, USA). Anion-exchange HPLC was performed using a TSKgel DEAE-5PW column (0.75×7.5 cm; Tosoh, Tokyo, Japan) at a flow rate of 1.0 mL/min [10]. The column was equilibrated with aqueous ammonia, pH 9.0. The aqueous ammonia, pH 9.0 was prepared by titration of water with ammonia solution. Five minutes after the sample had been injected, the concentration of ammonium acetate, pH 9.0, was increased linearly to 0.2 M in the first 20 min, and then to 0.5 M over the next 10 min. The PA-glycans were detected using a fluorescence spectrophotometer with an excitation wavelength of 310 nm and an emission wavelength of 380 nm. Size-fractionation HPLC was performed using a Waters Alliance HPLC system with a Waters 2475 fluorescence spectrophotometer (Milford, MA, USA). Size-fractionation HPLC was performed using a TSK gel Amide 80 column (0.46×7.5 cm; Tosoh, Tokyo, Japan) at a flow rate of 0.5 mL/min. The column was equilibrated with 50 mM ammonium formate, pH 4.4, containing 80% acetonitrile. The use of formate for the solvent allows the elution of carboxylate-containing PA-glycans under low salt concentration. After the sample had been injected, the acetonitrile concentration was decreased linearly from 80% to 65% over the first 5 min, 65% to 55% over the second 5 min, and then 55% to 30% over the next 25 min. The PA-glycans were detected using a fluorescence spectrophotometer with an excitation wavelength of 315 nm and an emission wavelength of 400 nm. The molecular size of each PA-glycan shown as *S* value is given in terms of glucose units based on the elution times of PA-IMO [11]. Briefly, the elution times of PA-IMO and the number of glucose residues were related with an approximate curve of quintic function. The glucose units of PA-glycans were calculated from the quintic function as a degree of glucose residues. Reversed phase HPLC was performed on a Cosmosil 5C18-P column (0.2×25 cm; Nacalai Tesque, Kyoto, Japan) at a flow rate of 0.2 mL/min. The column was equilibrated with 100 mM triethylamine acetate, pH 4.0, with 0.075% 1-butanol. Using triethylamine for the solvent results in a sharp elution profile of acidic PA-glycans. After injection of the sample, the 1-butanol concentration was increased linearly from 0.075% to 0.5% over 105 min. The PA-glycans were detected using an excitation wavelength of 315 nm and an emission wavelength of 400 nm. The retention time of each PA-glycan was converted to an *R* value by the reversed phase scale as described previously [7]. A conversion curve from the elution time to *R* value was drawn by using the retention times of eight standard PA-glycans, i.e., GN, GNF6, GN2, GN2F6, M3B, M3BF6, BIBS, and BIBSF6, as the most interior α1-6-linked fucose residues equally contributed to the *R* value. Structures and abbreviations of the PA-glycans are listed in Table S1.

### Preparation of the eight PA-glycan-standards for the reversed phase scale

GN and GN2 were prepared by pyridylamination from GlcNAc and *N,N′*-diacetylchitobiose, respectively, which were purchased from Wako Pure Chemical Industries, Inc. (Osaka, Japan). GNF6 and GN2F6 were prepared by pyridylamination from Fucα1-6GlcNAc and GlcNAcβ1-4(Fucα1-6)GlcNAc, respectively, which were chemically synthesized by Tokyo Chemical Industry Co., Ltd. (Tokyo, Japan). M3B was prepared from quail ovomucoid as described elsewhere [12]. M3BF6 was from squid skin as reported before [13]. BIBS and BIBSF6 were from bovine γ-globulins (Sigma-Aldrich, St. Louis, MO).

### Preparation of PA-glycans from human serum

Pooled normal human serum was purchased from Gemini Bio-Products (West Sacramento, CA, USA). *N*-Glycans were liberated from the glycoproteins of lyophilized serum by hydrazinolysis as described previously [11]. Briefly, 0.1 mL of the serum was lyophilized and then heated at 100°C for 10 h with 1 mL of anhydrous hydrazine. After removal of hydrazine by repeated evaporations, the glycans were re-*N*-acetylated with acetic anhydride in a saturated sodium bicarbonate solution, and then passed through a Dowex 50Wx2 (H^+^) cation exchanger (Dow Chemicals, Midland, MI, USA) to remove sodium ions. The reducing ends of the liberated glycans were tagged with the fluorophore 2-aminopyridine. Lyophilized samples were heated at 90°C for 60 min with 100 µL of pyridylamination reagent, and then heated at 80°C for 35 min after the addition of 350 µL of reducing reagent.

Purification of PA-glycans from the reaction mixture was performed as described before [10]. The reaction mixture was diluted with 0.75 mL of water and extracted twice using 1 mL of phenol/chloroform (1∶1 v/v) to remove the excess reagents. The water layer that contained the PA-glycans was purified by gel filtration on a column (1.5×18 cm, TSK-gel Toyopearl HW-40F, Tosoh, Tokyo, Japan) equilibrated with 10 mM ammonium acetate, pH 6.0. After loading the sample, the eluate between 10 and 25 mL was collected as the PA-glycan fraction. The PA-glycans were further purified using a graphite carbon cartridge (GL-Pak Carbograph 300 mg; GL Sciences Ltd, Tokyo, Japan). Salt concentration of the glycan mixture was adjust to 50 mM with ammonium acetate, pH 6.0, and loaded onto the cartridge. After washing with 5 mL of 50 mM ammonium acetate, pH 6.0, the glycans were eluted using 5 mL of 60% acetonitrile in the ammonium acetate buffer. The eluate was concentrated by a vacuum concentrator and dried by lyophilization.

### Enzyme digestions

PA-glycans (about 1 pmol) were digested with 40 mU of sialidase (*Arthrobacter ureafaciens*) in 20 µL of 0.1 M sodium acetate, pH 5.0, at 37°C for 18 h, or with 150 mU of α2-3sialidase in 20 µL of 0.05 M sodium acetate, pH 5.5, at 37°C for 20 min. The enzymes were inactivated by heating at 95°C for 3 min.

### MS analysis

PA-glycans separated by HPLC were lyophilized to remove volatile salts in the buffer, and 1–10 pmol were co-crystalized with 1 mg/mL of 2,5-dihudroxybenzoic acid in 30% ethanol on an AnchorChip target plate (Brucker Daltonics, Bllerica, MA, USA) according to the manufacturer's protocol. MALDI-TOF mass spectra were recorded using an Autoflex II (Brucker Daltonics) in reflector mode.

## Results

### Preparation of two-dimensional map of *N*- and *O*-glycans

**Figure 1:**
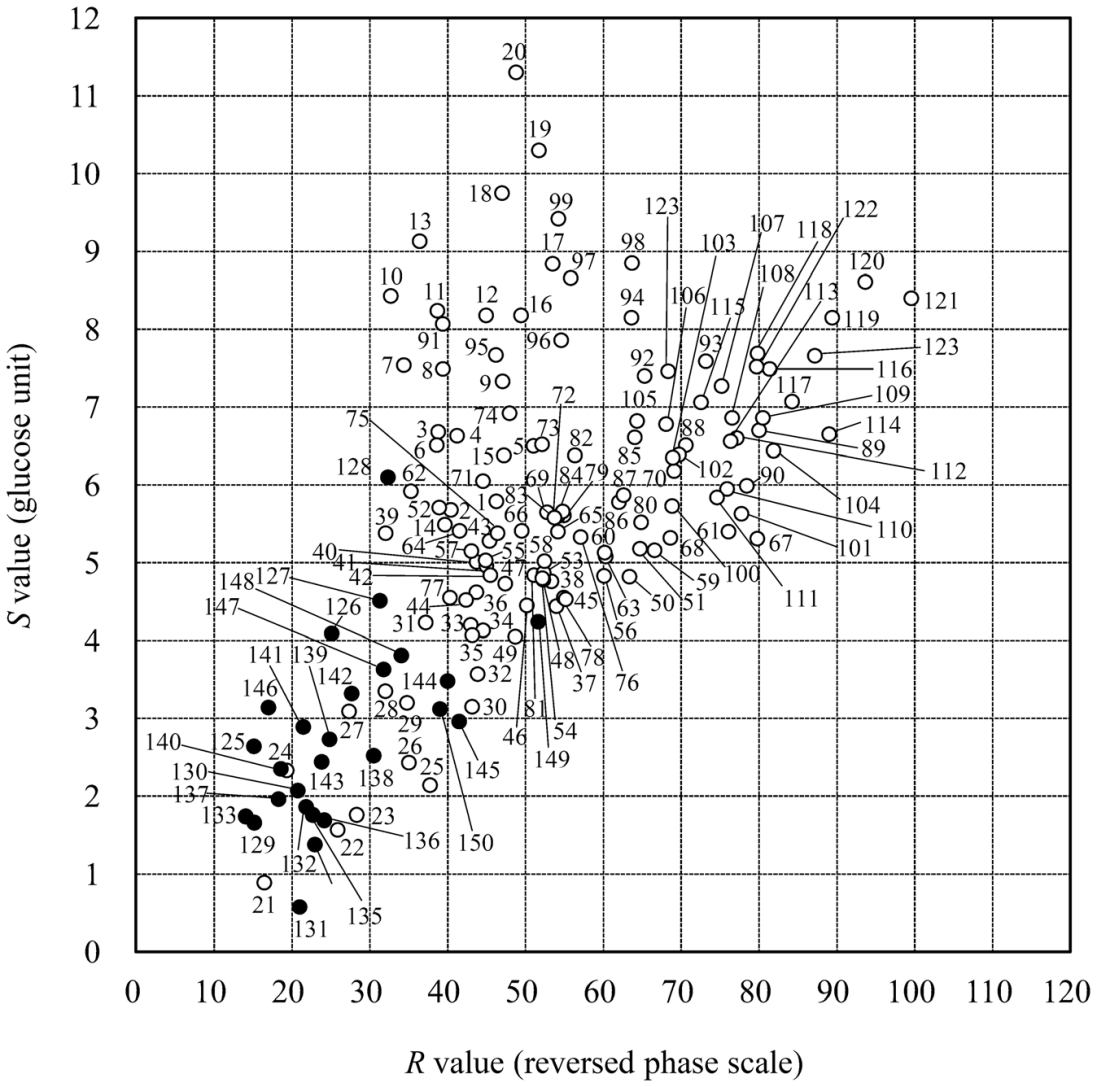
Glycan map of 150 standard glycans. White and black circles are *N*-linked and *O*-linked glycans, respectively. Data used for the plotting are listed in Table S1.

The 150 PA-glycans listed in Table S1 were chromatographed on two kinds of HPLC. The elution times of the PA-glycans on size-fractionation HPLC were converted to *S* values by glucose unit and those on reversed phase HPLC were converted to *R* values by using a reversed phase scale as described in the Materials and Methods section. The PA-glycans were plotted on a two-dimensional map with the *R* values as the horizontal axis and the *S* value as the vertical axis (Fig. 1). Those PA-glycans included *N*- and *O*-glycans with and without sialic acid. Most of them were divided well on the map. Although several glycans were not completely separated, simple MS analysis or enzyme digestion could easily distinguish them from each other. The characteristic values listed in Table S1 could be used as a database for identification of the PA-glycans. High reproducibility of *R* and *S* values was already verified in previous study [7], [10]. Those results indicate that the glycan-map prepared in this study is useful for comprehensive comparative analysis of the glycans.

### Empirical additivity rule of sugar residues

**Figure 2:**
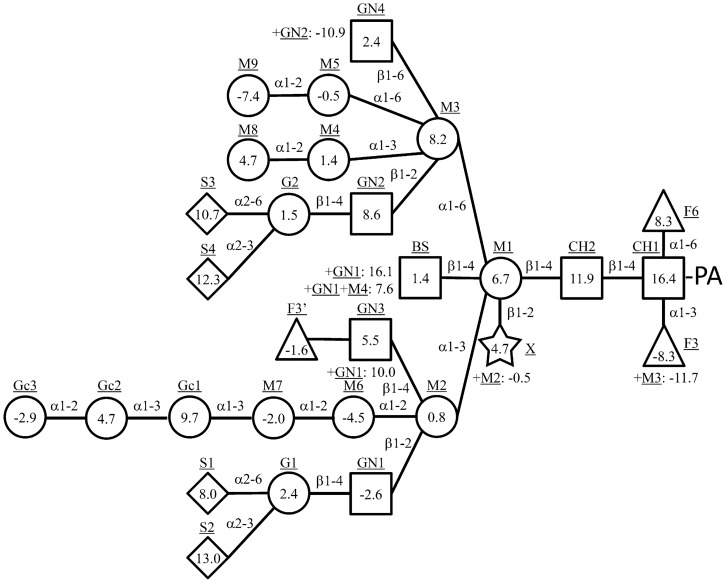
Partial elution times of sugar residues of *N*-glycan on a reversed phase HPLC. Partial elution time of each residue is written using symbols for the different types of monosaccharides. Circles indicate hexose, squares are GlcNAc, triangles are Fuc, the star is Xyl, and diamonds are NeuAc. Underlined letters are the residues, and the type of monosaccharides are followed: GN, GlcNAc; M, Man; G, Gal; X, Xyl; F, Fuc; Gc, Glc; S, NeuAc; BS, bisecting GlcNAc; CH, GlcNAc in *N,N′*-diacetylchitobiose core. The residues, X, GN3, GN4, and GN5 have two or three partial elution times corresponding to another specific residue. For example, the partial elution time of residue X is 4.7 when M2 residue exists and −0.5 when M2 is absent.

**Figure 3:**
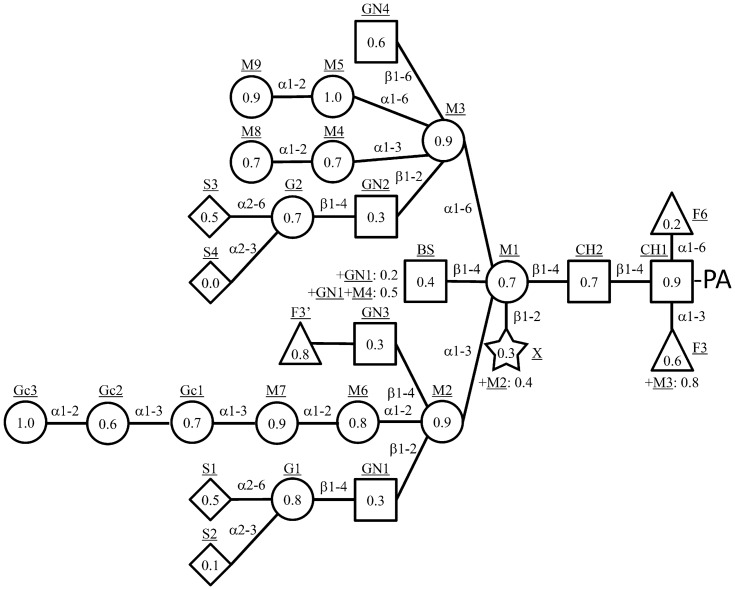
Partial elution times of sugar residues of *N*-glycan on a size-fractionation HPLC. The partial elution time of each residue is written using symbols for the different types of monosaccharides as the same manner in Figure 2.

The empirical additivity rule is based on the observation that the contribution to the elution time on reversed phase HPLC of a certain monosaccharide addition to a PA-glycan is scarcely influenced by other sugar residues [8], [9]. Their contribution degree was evaluated as a partial elution time that is specific to a position and kind of monosaccharides. The degree of the contribution made by each residue was calculated as the mean value of the differences between a pair of glycans with and without the residue under the new analytical condition. Data of difference and variance of the differences were listed in Table S2. The partial elution times obtained were indicated schematically in Figure 2. Some residues had two or three values of partial elution time. The xylose residue, shown as an X in Figure 2, took a value of 4.7 or −0.5 in the absence or presence of the M2 residue, respectively. On the other hand, the GlcNAc residue of GN3, so-called bisecting GlcNAc, took a value of 1.4 in the absence of GN1 and M4 residues, and values of 16.1 or 7.6 in the presence of GN1 or both GN1 and M4, respectively. The GN3 residue took values of 5.5 or 10.0 in the absence or presence of the GN1 residue, respectively. The GN4 residue took values of 2.4 or −10.9 in the absence or presence of the GN2 residue, respectively. Those splittings of the partial elution time might have been due to the interaction between the two residues. Actually, a previous NMR experiment showed the possibility of an interaction between the GN2 and GN4 residues [14]. Similarly, the partial elution time on size-fractionation HPLC was also calculated as glucose unit. Data of the differences and variance of the differences are listed in Table S2. The partial elution times obtained are indicated schematically in Figure 3. In this time we determined the partial elution times of sugar residues only on *N*-glycans, because there was not enough *O*-glycan data to obtain the partial elution times. The *R* values and *S* values of 107 PA-*N*-glycans calculated from the partial elution times are listed in Table S1. The calculated *S* values of 95 PA-*N*-glycans coincided with the measured *S* values with an error rate of less than 5%. The error rates of the calculated *S* values were more than 10% only in the case of three PA-*N*-glycans, GNF6, GN2, and GN2F6. The calculated *R* values of 92 PA-*N*-glycans coincided with the measured *R* values with an error rate of less than 5%. The error rates of calculated *R* values were more than 10% only in the case of three PA-*N*-glycans, AG1F6-M3, MO1-M3, and MO1F6-M3. This result might suggest that the M3 residue interact with the branch extended from the M2 residue.

### Mapping of human serum *N*- and *O*-glycans

**Figure 4:**
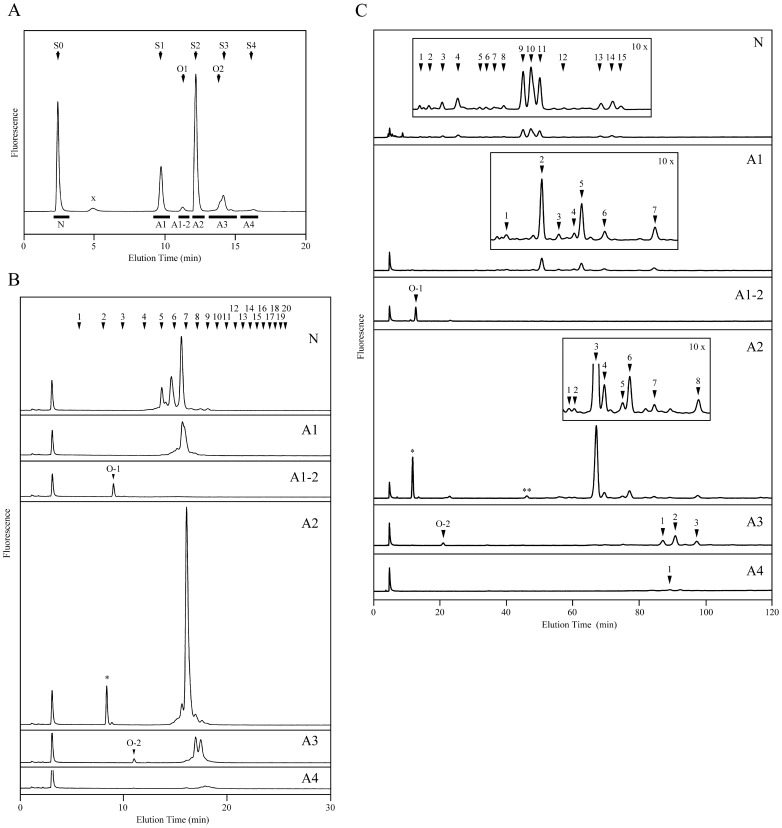
Separation of PA-glycans from human serum by three kinds of HPLCs. (a) Elution profile of DEAE anion-exchange-chromatography. PA-*N*-glycans from human serum were separated into neutral (N) and acidic (A1–A4) fractions. The thick bar indicates the collected fractions. X indicates a peak derived from reagents. Arrows labeled S0–S4 indicate elution positions of standard PA-*N*-glycans with 0–4 sialic acids, respectively. Arrows O1 and O2 are those of 03N-core1 and 36N-core1, respectively. (b) Elution profile of size-fractionation HPLC. The fractions isolated by DEAE chromatography were separated further by size-fractionation HPLC, respectively. Arrowheads indicate the elution positions of PA-isomalto-oligosaccharides with degrees of polymerization from 1 to 20. Asterisked peak is an artificial glycan that details are mentioned in the text. (c) Elution profile of reversed-phase HPLC. The fractions isolated by DEAE chromatography were separated further by reversed phase HPLC, respectively. The insets labeled 10× are magnified 10-fold on vertical axis. Arrowheads indicate collected peaks. Double asterisked peak indicates a peak that is epimerization product from 66N-BI (peak A2-3).

**Figure 5:**
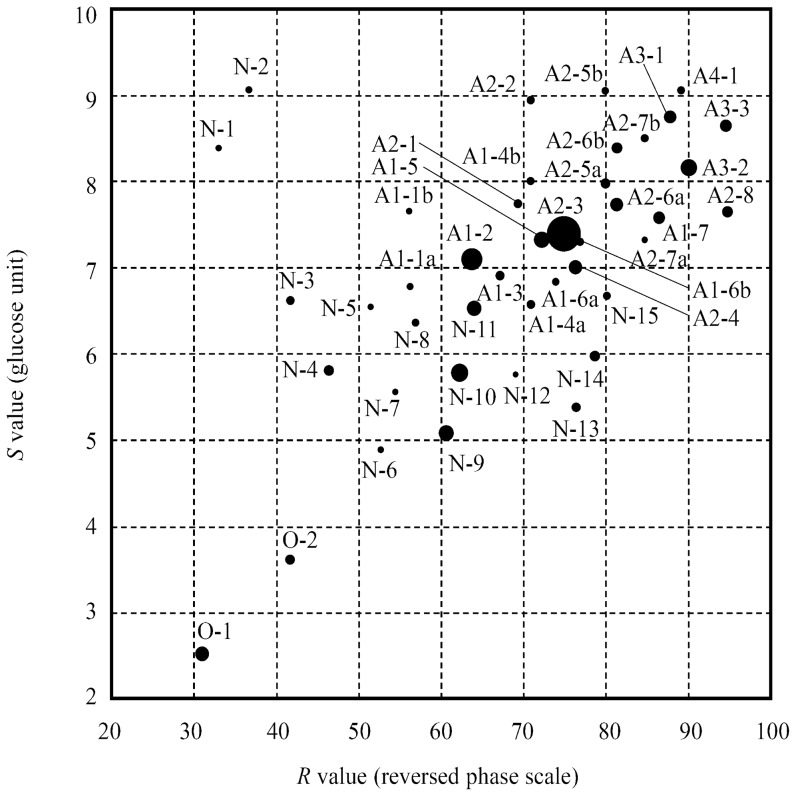
Two-dimensional HPLC map of the human serum PA-glycans. The retention times on size-fractionation and reversed phase HPLC were converted to *S* and *R* values, respectively, as described in Materials and methods. Diameter of the circles is proportional to the cubic root of the detected amount of PA-glycans. Therefore, the quantity ratio of 100 times is approximately equal to the diameter ratio of 4.6 times. Data used for the plotting are listed in Table S3.

PA-glycans were prepared from pooled normal human sera and sequentially separated by three kinds of HPLC. First, PA-glycans were separated to N, A1, A1-2, A2, A3, and A4 fractions by acidity with DEAE anion-exchange HPLC (Fig. 4a). The elution positions of the fraction N, A1 A2, A3, and A4 were corresponding to those of standard PA-*N*-glycans that bore 0 to 4 sialic acid, respectively. Small size sialyl PA-glycans were eluted somewhat later than large size sialyl PA-glycans on the DEAE HPLC. The elution position of the fraction A1-2 appeared following A1 was corresponding to that of 03N-core1. The fractions were further separated by size-fractionation and reversed phase HPLCs (Fig. 4b, c). The small size PA-glycans (O-1 and 2), which were probably *O*-glycans, were collected on size-fractionation HPLC (Fig. 4b). The peak labeled asterisk in Figure 4b was estimated as Siaα2-3Gal-PA, which was derived from a peeling product of sialyl core1 glycan, by further analysis (data not shown). Fractions O-1 and 2 were further separated by reversed phase HPLC. Their elution times were compared with the standard PA-glycans to estimate their structures (Table S3 and S4). As shown in Figures 4b and c, reversed phase HPLC achieved much better separability than size-fractionation HPLC to the large size PA-glycans. Then, we chose the reversed phase HPLC as a second separation of those PA-glycans. The peaks appeared on the reversed phase HPLC were collected and further separated by size-fractionation HPLC (data not shown). The quantity ratio of the isolated PA-glycans was accounted from their peak areas. The 42 PA-glycans which amounts were more than 0.4% to that of the most abundant species, A2-3 were collected as O-1, O-2, N1 to N15, A1-1a to A1-7, A2-1 to A2-8, A3-1 to A3-3, and A4-1 and further analyzed their structures (Fig. 4 and Table S3). The retention times of the isolated PA-*N*-glycans on reversed phase and size-fractionation HPLCs were converted to *R* and *S* values as described in Table S3 and plotted on two-dimensional glycan map (Fig. 5). The amounts of the PA-glycans were indicated as size of their dots. There were artificial C2-epimerization products, for example, double asterisk peak in Figure 4c, which was estimated as C2-epimer of reducing end GlcNAc from 66N-BI. That had the same molecular size with 66N-BI and its reducing end was *N*-acetylmannosamine (data not shown). Molar ratio of the epimerization product to its origin, 66N-BI was 2.3%.

### Structure analysis of neutral PA-glycans

The *R* and *S* values of the neutral PA-glycans, N1 to N15 were compared with data of the standard PA-glycans listed in Table S1. The values of all species other than N-12 accorded with those of one or two standard PA-glycan(s) (Table S3). Although the values of N-12 did not coincide with those of the standards, those showed good accordance with the values of BIBS-G1 and BIBS-G2 that inferred from the values of BIBS by using partial elution times of an empirical additivity rule. The abbreviations of PA-glycans were listed in Table S1 and S4. Since N-7 was closely scored on the map with two standards, BI-G1 and BI-G2, they were successively analyzed by reversed phase HPLC. As a result, N7 coincided with BI-G2 rather than BI-G1. N-10, N-12, and N-14 were closely scored on the map with each two isomers, BIF6-G1 and BIF6-G2, BIBS-G1 and BIBS-G2, BIBSF6-G1 and BIBSF6-G2, respectively. However, HPLC condition using in this study could not clearly separate those isomers. N-5 was very closely scored with M6C that was a rare isomer. To confirm that correspondence, N-5 was analyzed by MALDI-TOF MS. The measured m/z 1475.30 was compatible to the composition Hex_6_HexNAc_2_-PA (m/z 1475.51). From those observations, the structures of N-1–15 were proposed as M8A, M9A, M6B, M5A, M6C, AG12, BI-G2, BI, AG12F6, BIF6-G, BIF6, BIBS-G, AG12BSF6, BIBSF6-G, and BIBSF6, respectively (Table S4).

### Structure analysis of monosialo PA-*N*-glycans

**Figure 6:**
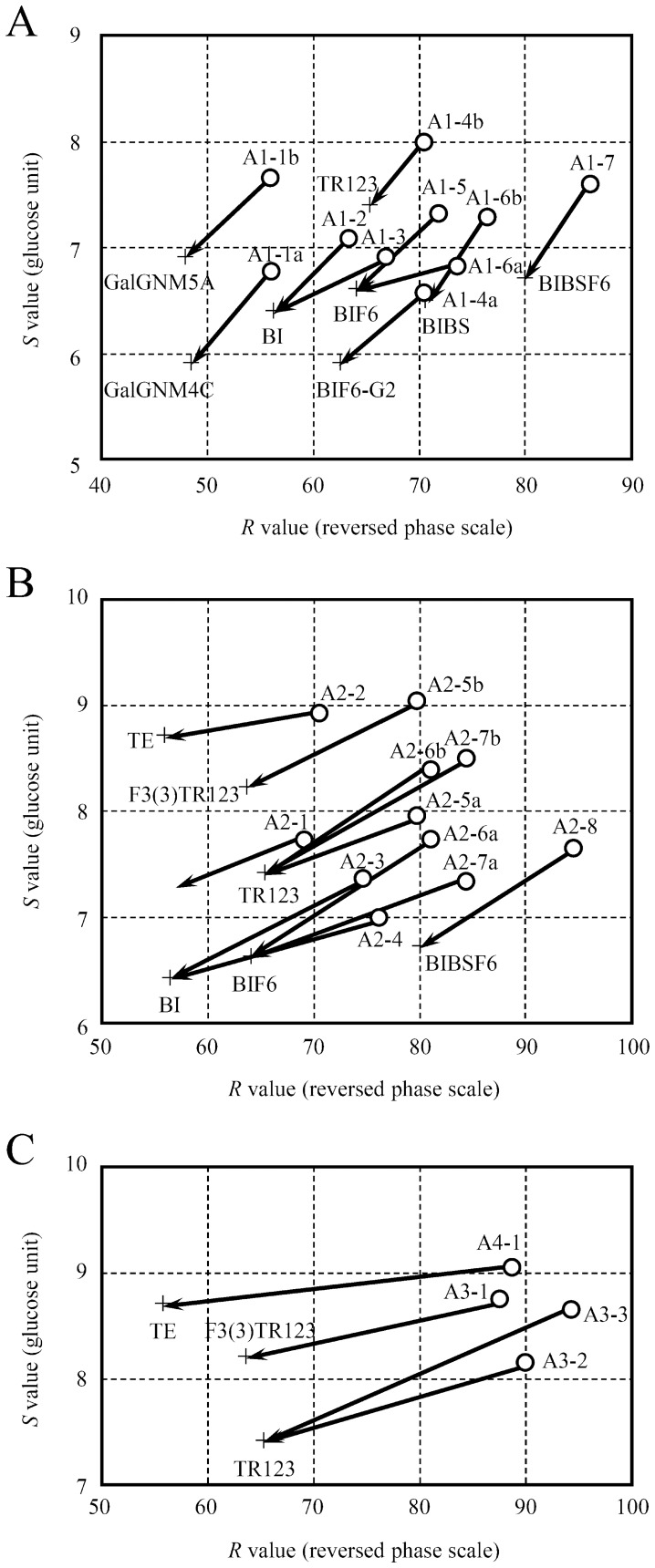
Sialidase digestion of the human serum PA-glycans. Plotting method is the same as Figure 5. Shifting of elution position of PA-glycans by *Arthrobactor* sialidase digestion are shown by arrows. Crosses are position of standard PA-glycans, which abbreviations are listed in Table S1. (a) A1 (monosialo), (b) A2 (disialo), (c) A3 (trisialo) and A4 (tetrasialo) PA-glycans.

From the results of comparison on the glycan map, the values of A1-2, A1-3 and A1-5 were according to 06N-BI, 60N-BI and 06N-BIF6, respectively. A1-1a, A1-1b, A1-4a, A1-6a, A1-6b, and A1-7 were closely scored with 6N-GalGNM4C, 6N-GalGNM5A, 06N-BIF6-G2, 60N-BIF6, 06N-BIBS and 06N-BIBSF6, respectively, that inferred by using the partial elution times. Those correspondences were confirmed by *Arthrobacter* sialidase digestion (Fig. 6a and Table S5). *Arthrobactor* sialidase liberated sialic acid from all of those monosialyl PA-*N*-glycans, whereas α2-3sialidase could not remove significantly under used condition. Since the map position of A1-4b was shifted to the position of TR123 by the sialidase digestion, it was estimated as 6N-TR123. From those observations, the structures of A1-1a, A1-1b, A1-2, A1-3, A1-4a, A1-4b, A1-5, A1-6a, A1-6b, and A1-7 were proposed as 6N-GalGNM4C, 6N-GalGNM5A, 06N-BI, 60N-BI, 06N-BIF6-G2, 6N-TR123, 06N-BIF6, 60N-BIF6, 06N-BIBS, and 06N-BIBSF6, respectively (Table S4).

### Structure analysis of disialo PA-glycans

**Figure 7:**
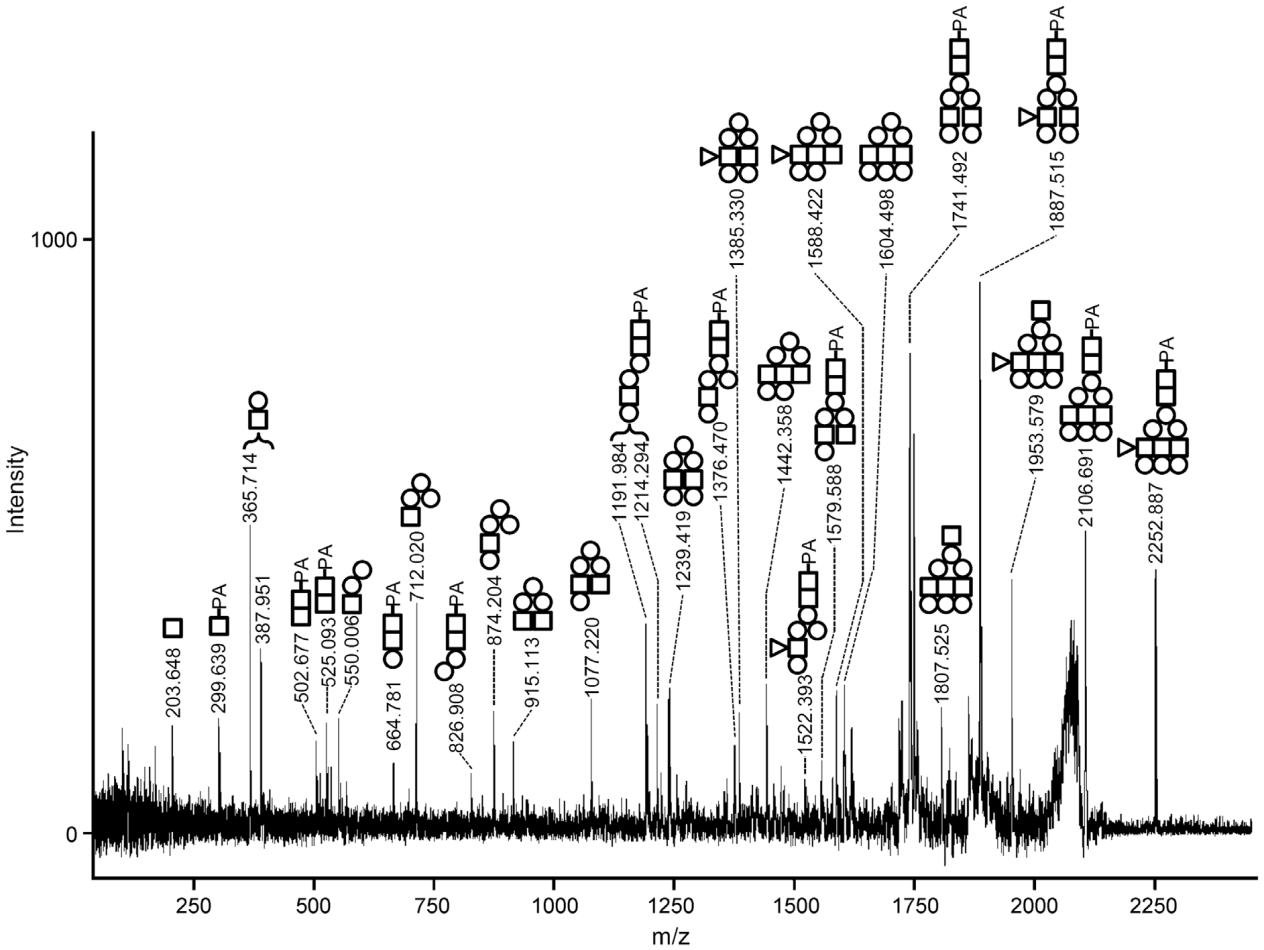
MS/MS analysis of desialylated A2-5b. Parent ion (*m/z* 2252.887) was subjected to positive-mode MS/MS. Selected characteristic fragment ions are annotated and compatible structures are shown. Circles indicate hexose, and squares are *N*-acetylhexosamine. The fragment ions including pyridylamino residue are estimated to be derivatives from reducing end.

From the results of comparison on the glycan map, the values of A2-3, A2-4, A2-5a, A2-6a, and A2-7a were according to 66N-BI, 36N-BI, 063N-TR123, 66N-BIF6, and 36N-BIF6, respectively. A2-8 was closely scored with 66N-BIBSF6 that inferred from the partial elution times. Those correspondences were confirmed by *Arthrobacter* sialidase digestion (Fig. 6b and Table S5). The map positions of the A2-4, A2-5b, A2-6b, A2-7b, and A2-8 digests coincided with those of BI, F3(3)TR123, TR123, TR123, and BIBSF6, respectively. Five PA-glycans, A2-1, A2-2, A2-4, A2-5a, and A2-7a were susceptible to α2-3sialidase digestion, and released single sialic acid. Thus, they may have both α2-3 and α2-6-linked sialic acids. To confirm the core structure of A2-5b, MS/MS analysis was carried out to a sialidase digest of A2-5b (Fig. 7). There were compatible fragments with composition dHex_1_Hex_6_HexNAc_4_ (*m/z* 1953.579), dHex_1_Hex_5_HexNAc_3_ (*m/z* 1588.422), and dHex_1_Hex_5_HexNAc_2_ (*m/z* 1385.330) which did not include a reducing end PA-GlcNAc. This observation supported that a fucose linked to a non-reducing end branch of A2-5b. A2-1 was susceptible to sialidase digestion, but the digest did not correspond to any standard PA-glycans we had (Fig. 6b). Although TR124 had near position to the digest, successive analysis of them by HPLC did not support their identity. We could not analyze further because of small amount of A2-1. From those observations, the structures of A2-2, A2-3, A2-4, A2-5a, A2-5b, A2-6a, A2-6b, A2-7a, A2-7b, and A2-8 were proposed as, (36)N-TE, 66N-BI, 36N-BI, 063N-TR123, diN-F3(3)TR123, 66N-BIF6, diN-TR123, 36N-BIF6, diN-TR123, and 66N-BIBSF6, respectively (Table S4).

### Structure analysis of tri and tetrasialo PA-glycans

From comparison on the glycan map, the values of A3-2 and A3-2 were according to 663N-TR123 and 666N-TR123, respectively. The map positions of the sialidase digests of A3-1, A3-2, A3-3, and A4-1 coincided with those of F3(3)TR123, TR123, TR123, and TE, respectively (Fig. 6c). A3-2 was susceptible to α2-3sialidase digestion and released single sialic acid, whereas A3-3 was not digested under used condition. This observation supported to the estimated structures of A3-2 and A3-3. The α2-3sialidase digestion of A3-1 and A4-1 did not give a clear result due to limited amounts of them. From those observations, the structures of A3-1, A3-2, A3-3, and A4-1 were proposed as triN-F3(3)TR123, 663N-TR123, 666N-TR123, and tetN-TE, respectively.

## Discussion

One of the most serious problems in the structure determination of glycans is the need to make fine distinctions among many isomers. Mass spectrometric analysis is not necessarily a decisive method for making such distinctions, because the assignment of fragment peaks is very difficult when unknown isomer mixtures are used. Generally, isomers must be separated before their structures can be analyzed. However, no single chromatography we examined offered sufficient separability of the various complex isomers of the glycans, so we used three successive HPLCs to isolate them. We improved the separation conditions sufficiently to analyze both *O*- and *N*-glycans with or without sialic acid in a lump. As a result, the glycan data could be displayed comprehensively on a single glycan map. Moreover, we reported here the mapping data of 150 PA-glycans and induced the partial elution times of *N*-glycans to enable the prediction of elution times on reversed phase and size-fractionation HPLCs. The partial elution time is useful for predicting the elution positions of isomers. For example, α1-6-linked and α1-3-linked core fucoses are easily distinguished, because their reversed phase partial elution times are +8.3 and −8.3, respectively. Moreover α2-3- and α2-6-linked *N*-acetylneuraminic acids are also distinguished easily because they have different partial elution times. Divergence of triantennary complex-type isomers is caused by the third GlcNAc residue attached to the AG12 biantennary core structure. In the even to such divergence, the reversed phase partial elution times of β1-2-linked GlcNAc on the α1-3Man branch and β1-6-linked GlcNAc on the α1-6-linked Man branch are +10.0 and −10.9, respectively. Therefore, since the isomers between the two groups are plotted remotely from each other on the map, it is easy to distinguish between them. We observed that *N*-glycolyl modification of sialic acid contributed negatively to the reversed phase partial elution time and positively to the size-fractionation partial elution time (unpublished observation). Those observations indicate the possibility that the mapping method could be used to validate of glycosylated biopharmaceuticals. Though we showed the mapping data of 26 *O*-glycans in this study, it is necessary to collect data on many other *O*-glycans to reliably determine partial elution times.

Glycan Atlas is a set of glycan maps over the whole body of an organism. We constructed it as an infrastructure for glycobiological study. We reported here a human serum glycan map, namely the first page of the human Glycan Atlas. We detected 42 kinds of PA-glycans of which contents were more than 0.4% against the most dominant species. Trace components include artificial substances, such as epimerized, acetylated, or peeled glycans. Epimerization occurs on glycans that have a bare reducing end in alkaline solution. It converts reducing end GlcNAc and GalNAc to *N*-acetylmannosamine and *N*-acetyltalosamine, respectively. The major process that causes epimerization is glycan liberation. About 2% of epimerization has arisen under the conditions we used. However, the *N*-glycan liberation by PNGase F digestion in weak alkaline solution accompanied an approximately 10-times-higher epimerization ratio than the method described in this report (unpublished observation). Artificial acetylation on glycans may occur in the reaction of re-*N*-acetylation by acetic anhydride. Its ratio is controlled to less than 1% of original glycans under the conditions we used (data not shown). The peeling reaction complicates the analysis of mucin-type *O*-linked glycans. The reducing end GalNAc is peeled off from glycans in alkaline solution when its 3-position is substituted. Siaα2-3Gal detected in this study is probably the artificial product of the peeling reaction of sialyl core 1 structures of mucin-type *O*-glycans. It is harder to induce a peeling reaction with anhydrous hydrazine than with strong alkaline aqueous solution, such as sodium hydroxide or concentrated ammonia solution, because water is necessary to proceed with the peeling reaction. However, a substantial amount of peeling product was still detected under the conditions we used in this study. To overcome that problem, it will be important to develop a controlled hydrazine treatment.

Human serum included a moderate amount of *O*-glycans that were a common type, sialyl core 1. There were a small amount of high mannose-type *N*-glycans, which probably derived from some glycoproteins including complement C3 [15], α2-macroglobulin [16], and immunoglobulin M [17]. Though two hybrid-type *N*-glycans, 6N-GalGNM4C and 6N-GalGNM5A, were also detected, it was unknown what kinds of glycoprotein they were derived from. Asialo biantennary complex-type *N*-glycans were found in moderate amounts. They were probably mainly derived from immunoglobulin G [18]. The dominant species were sialylated biantennary complex-type *N*-glycans. Especially, α2-6 sialylated biantennary species accounted for more than half of the PA-*N*-glycans from human serum. The most dominant one was 66N-BI, which accounted for over 40% of them. There were some sialylated tri- and tetra-antennary complex-type *N*-glycans. Notably, all of the triantennary glycans detected in this study had a β1-4GlcNAc branch on α1-3Man. This is one of the two types of dominant triantennary complex *N*-glycans; the other type has a β1-6GlcNAc branch on α1-6Man. The tetraantennary glycans observed in this study had both branching GlcNAc residues.

From the analysis of serum glycans, we acquired mapping data on 15 PA-*N*-glycans in addition to the 150 standard PA-glycans we had prepared for this study. The usefulness and accuracy of this mapping method will increase more and more as the mapping data accumulate by the analysis of other tissues and organs. Creation of the Glycan Atlas, which is a set of glycan maps for the whole body of an organism, is a work comparable to genome analysis, and is part of the infrastructure of glycobiology. We drew the first page of the Glycan Atlas in this report. In the future we will build an Internet database of the Glycan Atlas.

## Supporting Information

Table S1 Glycan structures and their mapping data.
(PDF)

Table S2 Partial elution times of sugar residues on *N*-glycan.
(PDF)

Table S3 Mapping data of PA-*N*-glycans from human serum.
(PDF)

Table S4 Proposed structures of human serum glycans.
(PDF)

Table S5 Sialidase digestions of PA-*N*-glycans from human serum.
(PDF)
