# Supplementary material for: Improved Method for Drawing of a Glycan Map, and the First Page of Glycan Atlas, Which Is a Compilation of Glycan Maps for a Whole Organism
Source: PLoS One. 2014 Jul 9;9(7):e102219. doi: 10.1371/journal.pone.0102219 (PMC4090225; doi:10.1371/journal.pone.0102219)
Supplement: Table S1 — Glycan structures and their mapping data. (PDF) [file pone.0102219.s001.pdf]

**Table S1. Glycan structures and their mapping data.**

| No. | Structure[1] | Abbreviation | *S* value measured[2] | *R* value measured[2] | *S* value calculated[3] | *R* value calculated[3] |
|---|---|---|---|---|---|---|
| 1 | 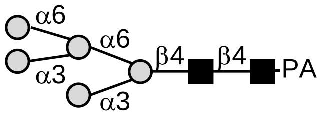  | M5A | 5.8 | 46.2 | 5.8 | 44.9 |
| 2 | 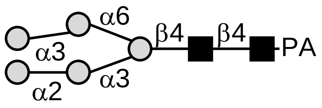  | M5C | 5.7 | 40.4 | 5.6 | 40.9 |
| 3 | 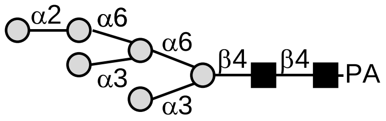  | M6A | 6.7 | 38.8 | 6.7 | 37.5 |
| 4 | 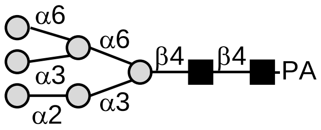  | M6B | 6.6 | 41.2 | 6.6 | 40.4 |
| 5 | 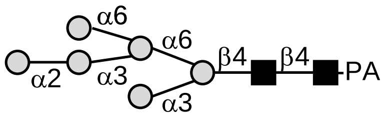  | M6C | 6.5 | 51.0 | 6.5 | 49.6 |

| | | | | | | |
|---|---|---|---|---|---|---|
| 6 | 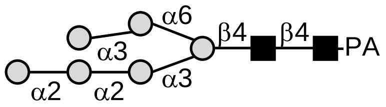 | M6E | 6.5 | 38.6 | 6.5 | 38.9 |
| 7 | 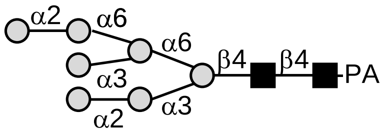 | M7A | 7.5 | 34.4 | 7.5 | 33.0 |
| 8 | 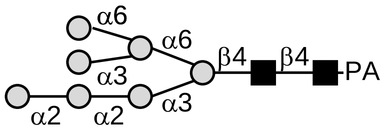 | M7B | 7.5 | 39.4 | 7.5 | 38.4 |
| 9 | 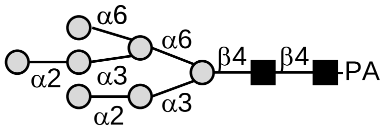 | M7D | 7.3 | 47.0 | 7.3 | 45.1 |
| 10 | 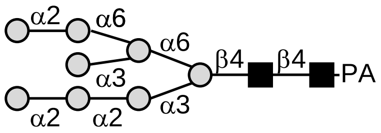 | M8A | 8.4 | 32.7 | 8.4 | 31.0 |
| 11 | 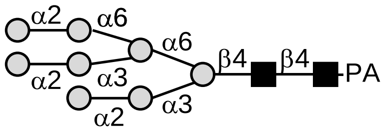 | M8B | 8.2 | 38.7 | 8.2 | 37.7 |
| 12 | 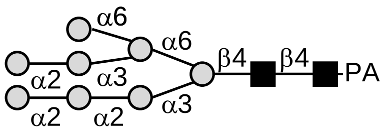 | M8C | 8.2 | 44.9 | 8.2 | 43.1 |

| | | | | | | |
|---|---|---|---|---|---|---|
| 13 | 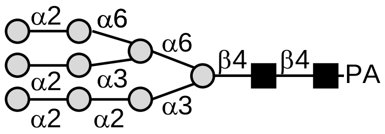 | M9A | 9.1 | 36.4 | 9.1 | 35.7 |
| 14 | 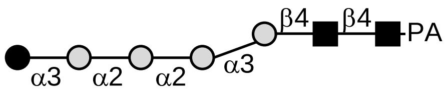 | G1M4E | 5.5 | 39.6 | 5.6 | 39.0 |
| 15 | 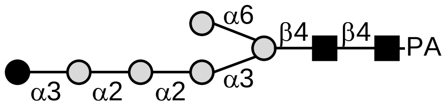 | G1M5B | 6.4 | 47.2 | 6.5 | 47.2 |
| 16 | 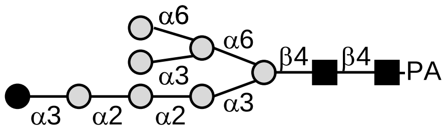 | G1M7B | 8.2 | 49.4 | 8.2 | 48.1 |
| 17 | 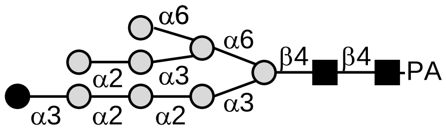 | G1M8C | 8.8 | 53.5 | 8.9 | 52.8 |
| 18 | 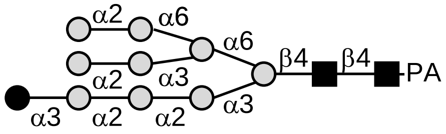 | G1M9A | 9.8 | 47.0 | 9.8 | 45.4 |
| 19 | 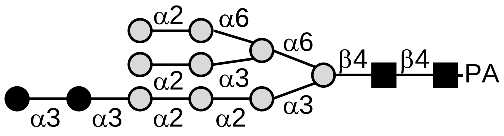 | G2M9A | 10.3 | 51.7 | 10.4 | 50.1 |

| | | | | | | |
|---|---|---|---|---|---|---|
| 20 | 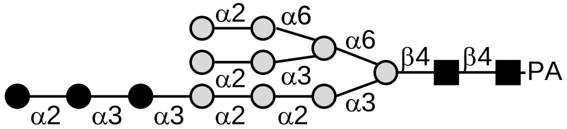 | G3M9A | 11.3 | 48.8 | 11.4 | 47.2 |
| 21 | 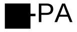 | GN | 0.9 | 16.4 | 0.9 | 16.4 |
| 22 | 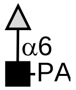 | GNF6 | 1.6 | 25.9 | 1.1 | 24.7 |
| 23 | 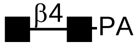 | GN2 | 1.8 | 28.3 | 1.6 | 28.3 |
| 24 | 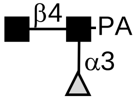 | GN2F3 | 2.3 | 19.3 | 2.2 | 20.0 |
| 25 | 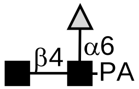 | GN2F6 | 2.1 | 37.7 | 1.8 | 36.6 |
| 26 | 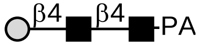 | M1A | 2.4 | 35.0 | 2.3 | 35.0 |
| 27 | 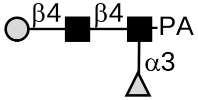 | M1AF3 | 3.1 | 27.3 | 2.9 | 26.7 |
| 28 | 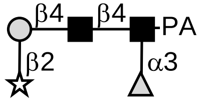 | M1AF3X | 3.4 | 32.0 | 3.2 | 31.4 |

| | | | | | | |
|---|---|---|---|---|---|---|
| 29 | 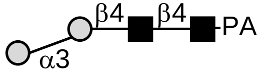 | M2A | 3.2 | 34.8 | 3.2 | 35.8 |
| 30 | 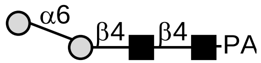 | M2B | 3.2 | 43.1 | 3.2 | 43.2 |
| 31 | 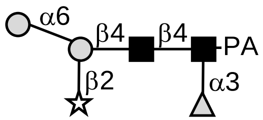 | M2BF3X | 4.2 | 37.2 | 4.1 | 39.6 |
| 32 | 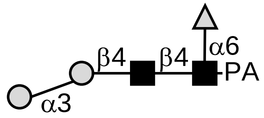 | M2AF6 | 3.6 | 43.8 | 3.4 | 44.1 |
| 33 | 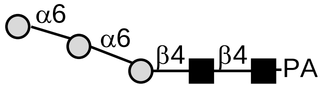 | M3A | 4.2 | 43.0 | 4.2 | 42.7 |
| 34 | 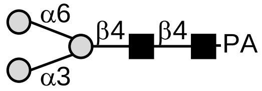 | M3B | 4.1 | 44.5 | 4.1 | 44.0 |
| 35 | 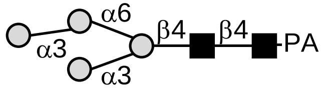 | M3C | 4.1 | 43.1 | 3.9 | 44.6 |
| 36 | 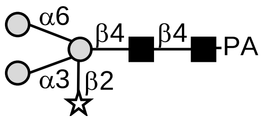 | M3BX | 4.6 | 43.7 | 4.4 | 43.5 |

| | | | | | | |
|---|---|---|---|---|---|---|
| 37 | 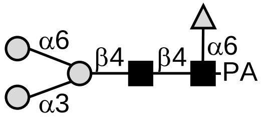 | M3BF6 | 4.4 | 54.0 | 4.3 | 52.3 |
| 38 | 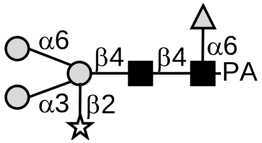 | M3BF6X | 4.8 | 53.3 | 4.7 | 51.8 |
| 39 | 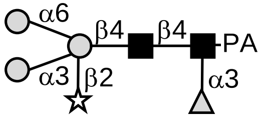 | M3BF3X | 5.4 | 32.0 | 5.2 | 31.8 |
| 40 | 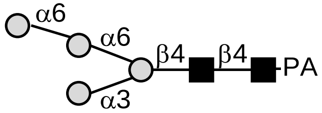 | M4A | 5.0 | 43.7 | 5.1 | 43.5 |
| 41 | 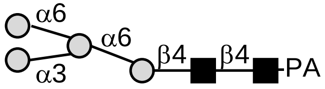 | M4B | 5.0 | 45.0 | 4.9 | 44.1 |
| 42 | 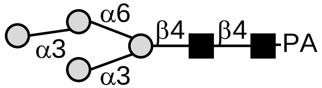 | M4C | 4.8 | 45.5 | 4.8 | 45.4 |
| 43 | 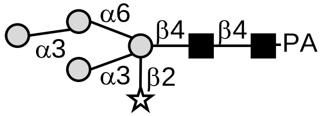 | M4CX | 5.3 | 45.4 | 5.2 | 44.9 |

| | | | | | | |
|---|---|---|---|---|---|---|
| 44 | 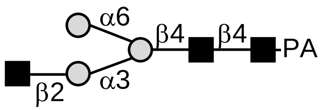 | AG1 | 4.5 | 42.4 | 4.4 | 41.4 |
| 45 | 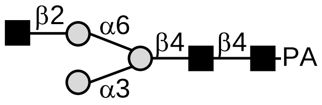 | AG2 | 4.5 | 55.2 | 4.4 | 52.6 |
| 46 | 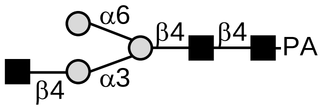 | AG3 | 4.5 | 50.2 | 4.4 | 49.5 |
| 47 | 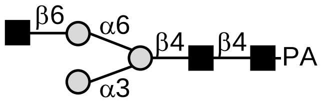 | AG4 | 4.7 | 47.4 | 4.7 | 46.4 |
| 48 | 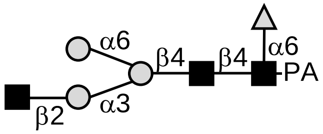 | AG1F6 | 4.8 | 52.3 | 4.6 | 49.7 |
| 49 | 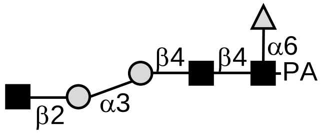 | AG1F6-M3 | 4.1 | 48.7 | 4.2 | 41.5 |
| 50 | 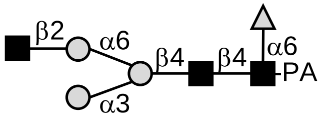 | AG2F6 | 4.8 | 63.4 | 4.9 | 60.9 |

| | | | | | | |
|---|---|---|---|---|---|---|
| 51 | 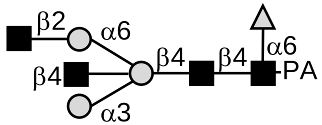 | AG2BSF6 | 5.2 | 64.7 | 5.3 | 62.3 |
| 52 | 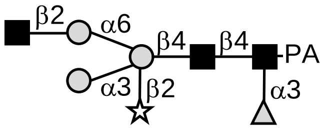 | AG2F3X | 5.7 | 38.9 | 5.5 | 40.4 |
| 53 | 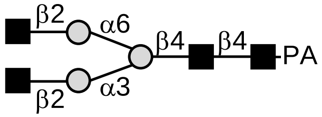 | AG12 | 4.9 | 52.3 | 4.7 | 50.0 |
| 54 | 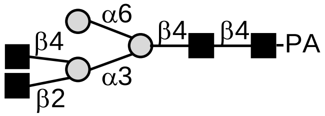 | AG13 | 4.8 | 52.2 | 4.7 | 51.4 |
| 55 | 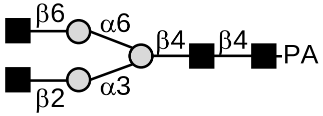 | AG14 | 5.0 | 44.9 | 5.0 | 43.8 |
| 56 | 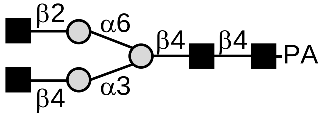 | AG23 | 4.8 | 60.1 | 4.7 | 58.1 |
| 57 | 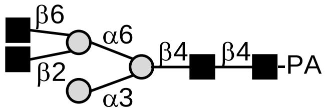 | AG24 | 5.2 | 43.0 | 5.0 | 41.7 |

| | | | | | | |
|---|---|---|---|---|---|---|
| 58 | 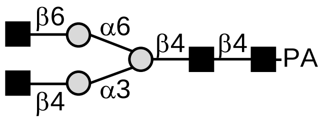 | AG34 | 5.0 | 52.4 | 5.0 | 51.9 |
| 59 | 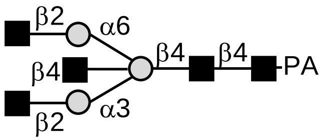 | AG12BS | 5.2 | 66.6 | 4.9 | 66.1 |
| 60 | 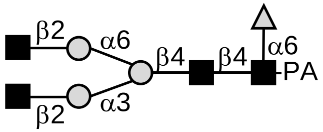 | AG12F6 | 5.1 | 60.3 | 4.9 | 58.3 |
| 61 | 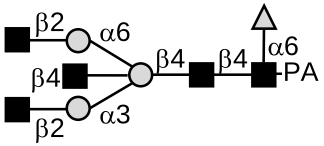 | AG12BSF6 | 5.4 | 76.1 | 5.1 | 74.4 |
| 62 | 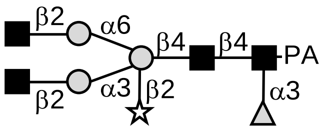 | AG12F3X | 5.9 | 35.3 | 5.8 | 37.8 |
| 63 | 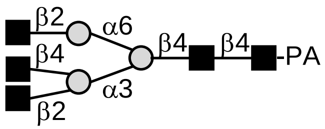 | AG123 | 5.1 | 60.2 | 5.0 | 60.0 |
| 64 | 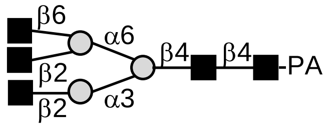 | AG124 | 5.4 | 41.5 | 5.3 | 39.1 |

| | | | | | | |
|---|---|---|---|---|---|---|
| 65 | 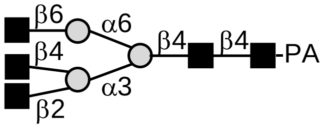 | AG134 | 5.4 | 54.2 | 5.3 | 53.8 |
| 66 | 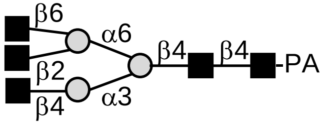 | AG234 | 5.4 | 49.5 | 5.3 | 47.2 |
| 67 | 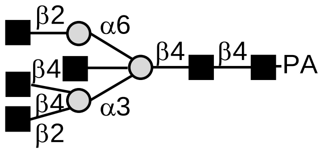 | AG123BS | 5.3 | 79.8 | 5.3 | 76.1 |
| 68 | 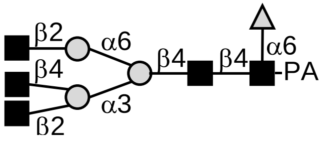 | AG123F6 | 5.3 | 68.6 | 5.2 | 68.3 |
| 69 | 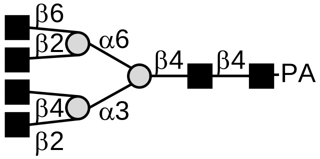 | AG1234 | 5.7 | 52.8 | 5.6 | 49.1 |
| 70 | 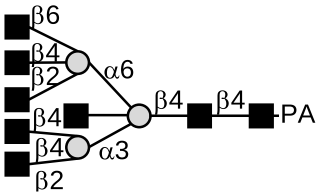 | AG12345BS | 6.2 | 69.1 | -[4] | -[4] |

| | | | | | | |
|---|---|---|---|---|---|---|
| 71 | 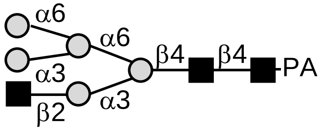 | GNM5A | 6.1 | 44.5 | 6.1 | 42.3 |
| 72 | 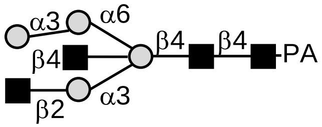 | GNM4CBS | 5.6 | 53.7 | 5.6 | 50.4 |
| 73 | 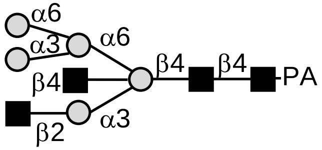 | GNM5ABS | 6.5 | 52.1 | 6.6 | 49.9 |
| 74 | 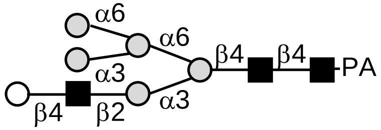 | GalGNM5A | 6.9 | 47.9 | 6.9 | 44.7 |
| 75 | 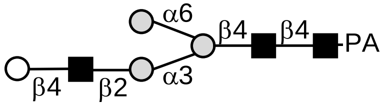 | MO1 | 5.4 | 46.4 | 5.2 | 43.8 |
| 76 | 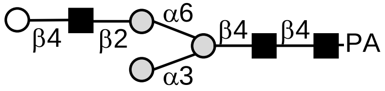 | MO2 | 5.3 | 57.1 | 5.1 | 54.1 |
| 77 | 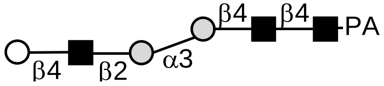 | MO1-M3 | 4.6 | 40.3 | 4.3 | 35.6 |
| 78 | 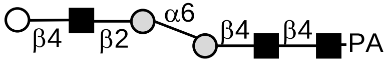 | MO2-M2 | 4.6 | 54.9 | 4.2 | 53.3 |

| | | | | | | |
|---|---|---|---|---|---|---|
| 79 | 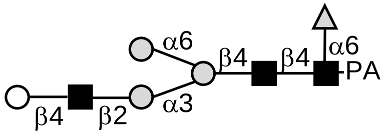 | MO1F6 | 5.6 | 54.9 | 5.4 | 52.1 |
| 80 | 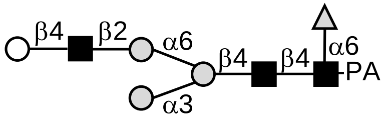 | MO2F6 | 5.5 | 64.8 | 5.3 | 62.4 |
| 81 | 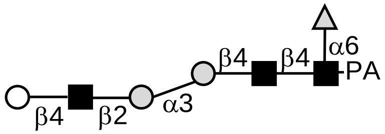 | MO1F6-M3 | 4.8 | 51.1 | 4.5 | 43.9 |
| 82 | 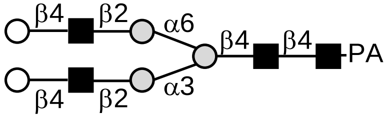 | BI | 6.4 | 56.3 | 6.2 | 53.9 |
| 83 | 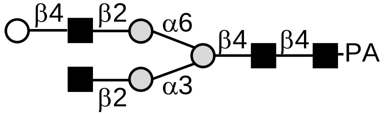 | BI-G1 | 5.6 | 54.1 | 5.4 | 51.5 |
| 84 | 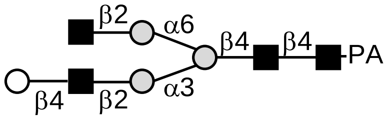 | BI-G2 | 5.7 | 54.8 | 5.5 | 52.4 |
| 85 | 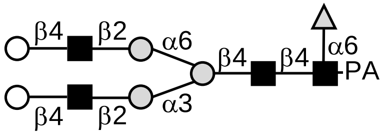 | BIF6 | 6.6 | 64.0 | 6.4 | 62.2 |

| | | | | | | |
|---|---|---|---|---|---|---|
| 86 | 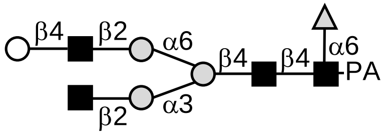 | BIF6-G1 | 5.8 | 62.0 | 5.6 | 59.8 |
| 87 | 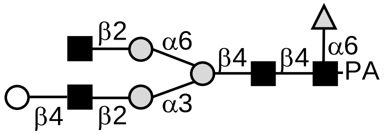 | BIF6-G2 | 5.9 | 62.6 | 5.7 | 60.7 |
| 88 | 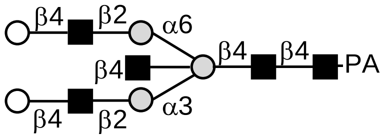 | BIBS | 6.5 | 70.6 | 6.4 | 70.0 |
| 89 | 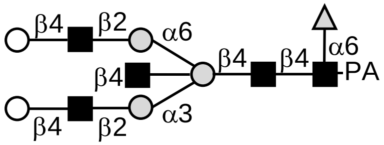 | BIBSF6 | 6.7 | 80.0 | 6.6 | 78.3 |
| 90 | 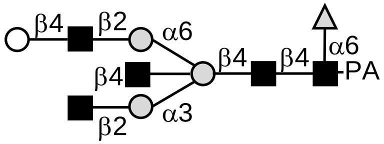 | BIBSF6-G1 | 6.0 | 78.4 | 5.8 | 75.9 |
| 91 | 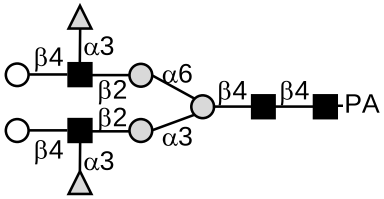 | diF3BI | 8.1 | 39.4 | - | - |

| | | | | | | |
|---|---|---|---|---|---|---|
| 92 | 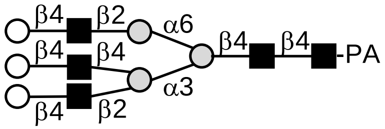 | TR123 | 7.4 | 65.3 | - | - |
| 93 | 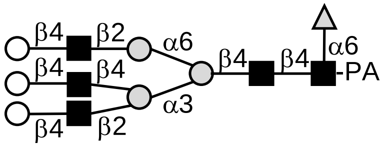 | TR123F6 | 7.6 | 73.2 | - | - |
| 94 | 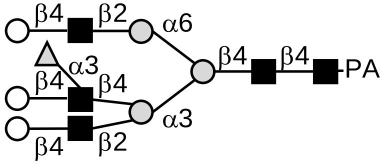 | F3(3)TR123 | 8.2 | 63.6 | - | - |
| 95 | 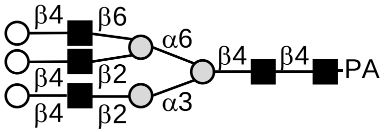 | TR124 | 7.7 | 46.2 | - | - |
| 96 | 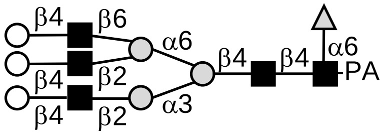 | TR124F6 | 7.9 | 54.6 | - | - |
| 97 | 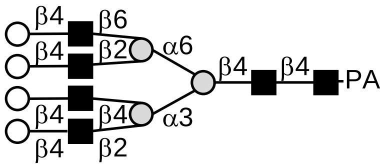 | TE | 8.7 | 55.8 | - | - |

| | | | | | | |
|---|---|---|---|---|---|---|
| 98 | 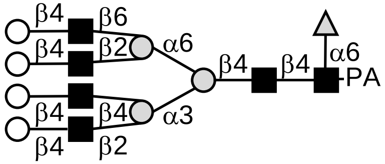 | TEF6 | 8.9 | 63.7 | - | - |
| 99 | 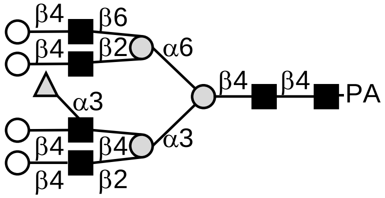 | F3(3)TE | 9.4 | 54.2 | - | - |
| 100 | 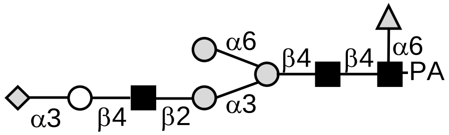 | 3N-MO1F6 | 5.7 | 68.8 | 5.5 | 65.1 |
| 101 | 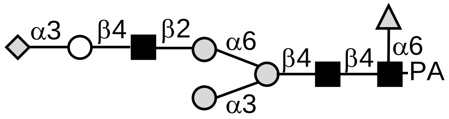 | 3N-MO2F6 | 5.6 | 77.7 | 5.3 | 74.7 |
| 102 | 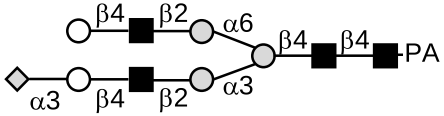 | 03N-BI | 6.4 | 69.7 | 6.3 | 66.9 |
| 103 | 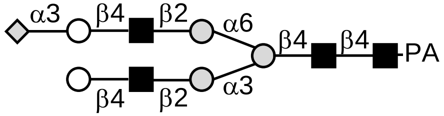 | 30N-BI | 6.4 | 68.9 | 6.2 | 66.2 |

| | | | | | | |
|---|---|---|---|---|---|---|
| 104 | 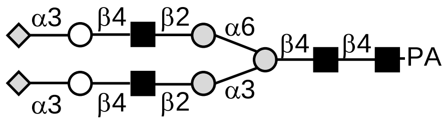 | 33N-BI | 6.4 | 81.9 | 6.3 | 79.2 |
| 105 | 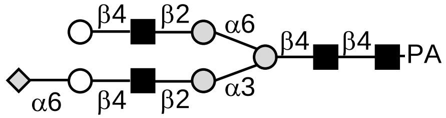 | 06N-BI | 6.8 | 64.3 | 6.7 | 61.9 |
| 106 | 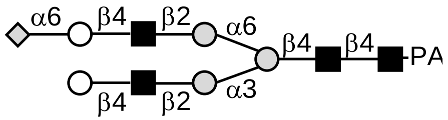 | 60N-BI | 6.8 | 68.1 | 6.7 | 64.6 |
| 107 | 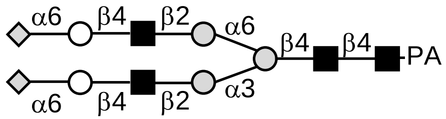 | 66N-BI | 7.3 | 75.2 | 7.2 | 72.6 |
| 108 | 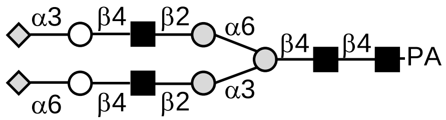 | 36N-BI | 6.9 | 76.5 | 6.7 | 74.2 |
| 109 | 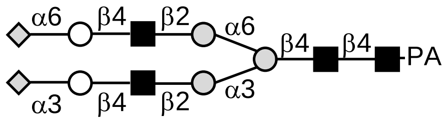 | 63N-BI | 6.9 | 80.5 | 6.8 | 77.6 |
| 110 | 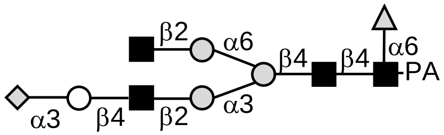 | 03N-BIF6-G2 | 6.0 | 75.9 | 5.8 | 73.7 |

| | | | | | | |
|---|---|---|---|---|---|---|
| 111 | 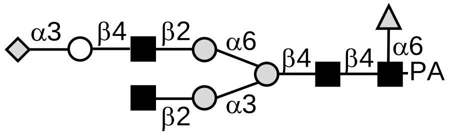 | 30N-BIF6-G1 | 5.8 | 74.6 | 5.6 | 72.1 |
| 112 | 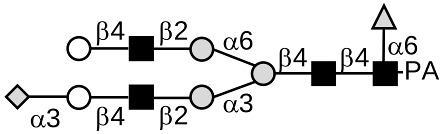 | 03N-BIF6 | 6.6 | 77.1 | 6.5 | 75.2 |
| 113 | 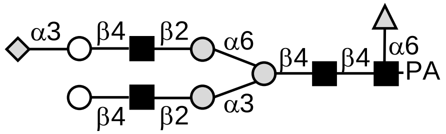 | 30N-BIF6 | 6.6 | 76.4 | 6.4 | 74.5 |
| 114 | 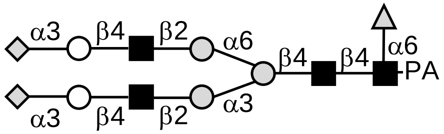 | 33N-BIF6 | 6.7 | 89.0 | 6.5 | 87.5 |
| 115 | 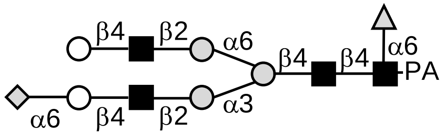 | 06N-BIF6 | 7.1 | 72.5 | 6.9 | 70.2 |
| 116 | 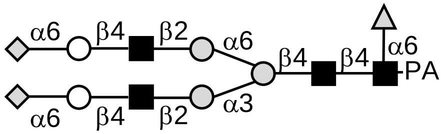 | 66N-BIF6 | 7.5 | 81.4 | 7.4 | 80.9 |
| 117 | 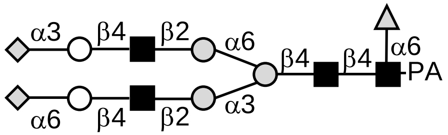 | 36N-BIF6 | 7.1 | 84.2 | 6.9 | 82.5 |

| | | | | | | |
|---|---|---|---|---|---|---|
| 118 | 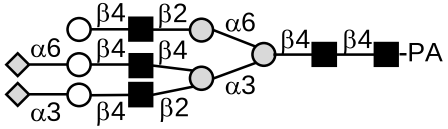 | 063N-TR123 | 7.7 | 79.8 | - | - |
| 119 | 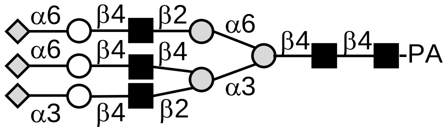 | 663N-TR123 | 8.2 | 89.4 | - | - |
| 120 | 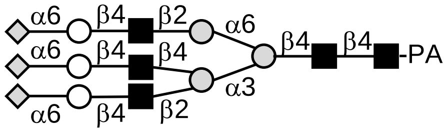 | 666N-TR123 | 8.6 | 93.7 | - | - |
| 121 | 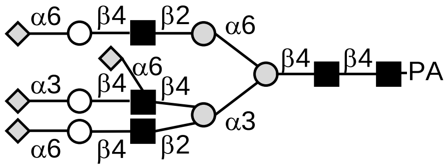 | 63(6)6N-TR123 | 8.4 | 99.6 | - | - |
| 122 | 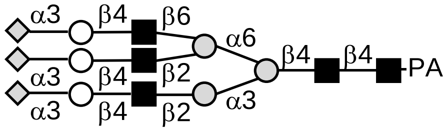 | 333N-TR124 | 7.5 | 79.7 | - | - |
| 123 | 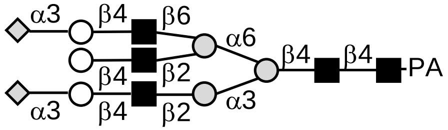 | 303N-TR124 | 7.5 | 68.3 | - | - |

| | | | | | | |
|---|---|---|---|---|---|---|
| 124 | 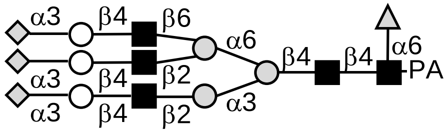  | 333N-TR124F6 | 7.7 | 87.2 | - | - |
| 125 | 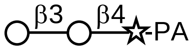  | Linkage3 | 2.6 | 15.1 | - | - |
| 126 | 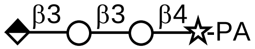  | Linkage4 | 4.1 | 25.1 | - | - |
| 127 | 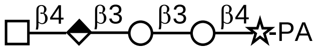  | Linkage5 | 4.5 | 31.3 | - | - |
| 128 | 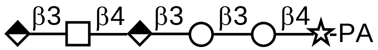  | Linkage6 | 6.1 | 32.3 | - | - |
| 129 | 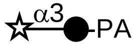  | XG | 1.7 | 15.1 | - | - |
| 130 | 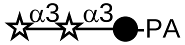  | X2G | 2.1 | 20.7 | - | - |
| 131 | 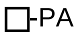  | Tn | 0.6 | 20.3 | - | - |
| 132 | 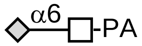  | 6N-Tn | 1.9 | 21.8 | - | - |
| 133 | 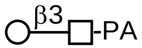  | core1 | 1.7 | 14.0 | - | - |

| | | | | | | |
|---|---|---|---|---|---|---|
| 134 | 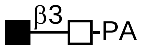 | core3 | 1.4 | 22.9 | - | - |
| 135 | 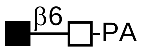 | core6 | 1.8 | 22.6 | - | - |
| 136 | 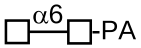 | core7 | 1.7 | 24.1 | - | - |
| 137 | 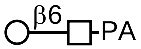 | core8 | 2.0 | 18.3 | - | - |
| 138 | 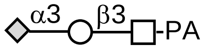 | 03N-core1 | 2.5 | 30.5 | - | - |
| 139 | 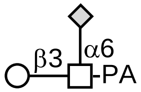 | 60N-core1 | 2.7 | 24.8 | - | - |
| 140 | 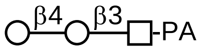 | Gb4core1 | 2.4 | 18.6 | - | - |
| 141 | 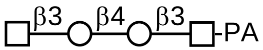 | GaNb3Gb4core1 | 2.9 | 21.4 | - | - |
| 142 | 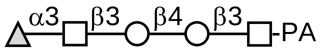 | F3GaNb3Gb4core1 | 3.3 | 27.7 | - | - |
| 143 | 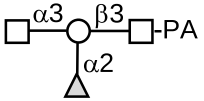 | GaNa3(F2)core1 | 2.4 | 23.8 | - | - |

| | | | | | | |
|---|---|---|---|---|---|---|
| 144 | 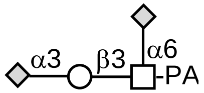 | 36N-core1 | 3.5 | 40.0 | - | - |
| 145 | 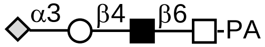 | 3N-Gb4core6 | 3.0 | 41.5 | - | - |
| 146 | 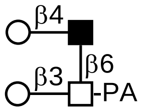 | Gb4core2 | 3.1 | 17.0 | - | - |
| 147 | 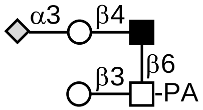 | 30N-Gb4core2 | 3.6 | 31.8 | - | - |
| 148 | 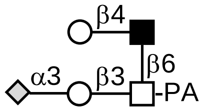 | 03N-Gb4core2 | 3.8 | 34.0 | - | - |
| 149 | 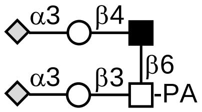 | 33N-Gb4core2 | 4.2 | 51.6 | - | - |
| 150 | 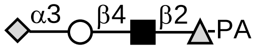 | 3N-Gb4GNb2F | 3.1 | 39.0 | - | - |

[1] Monosaccharide symbols are indicated as follows: white circle, Gal; white square, GalNAc; gray circle, Man; gray triangle, Fuc; black circle, Glc; black square, GlcNAc; star, Xyl; gray diamond, NeuAc; black and white diamond, GlcA.

[2] Converted values from actual measurements by elution times of standard glycans.
[3] Calculated values as the sum of partial elution times shown in Figures 2 and 3.
[4] Not calculated. Because the glycan included residue(s) of which partial elution time was not determined.
