# Supplementary material for: Improved Method for Drawing of a Glycan Map, and the First Page of Glycan Atlas, Which Is a Compilation of Glycan Maps for a Whole Organism
Source: PLoS One. 2014 Jul 9;9(7):e102219. doi: 10.1371/journal.pone.0102219 (PMC4090225; doi:10.1371/journal.pone.0102219)
Supplement: Table S2 — Partial elution times of sugar residues on N-glycan. (PDF) [file pone.0102219.s002.pdf]

Table S2. Partial elution times of sugar residues on *N*-glycan.

Residue[1]	Structure without the residue[2]	Structure with the residue	Δ*S* value[3]	Δ*R* value[4]
CH1-PA	-	GN	**0.89**[5]	**16.42**
CH2	GN	GN2	0.87	11.87
	GNF6	GN2F6	0.57	11.87
			**0.72** (Av)[6]	**11.87** (Av)
			0.30 (Di)[7]	0.00 (Di)
M1	GN2	M1A	**0.67**	**6.71**
M2	M1A	M2A	0.77	-0.24
	M2B	M3B	0.98	1.42
	M4B	M5A	0.83	1.29
			**0.86** (Av)	**0.82** (Av)
			0.11 (Sd)[8]	0.92 (Sd)
M2 with X	M2BF3X	M3BF3X	**1.15**	**-5.15**
M3	M1A	M2B	0.72	8.12
	M2A	M3B	0.93	9.78
	M2AF6	M3BF6	0.87	10.12
	G1M4E	G1M5B	0.89	7.58
			**0.85** (Av)	**8.90** (Av)
			0.09 (Sd)	1.24 (Sd)
M3 with F3	M1AF3X	M2BF3X	**0.88**	**5.18**
M4	M3A	M4B	0.76	0.41

	M3B	M4C	0.71	0.92
	M3BX	M4CX	0.66	1.69
	M4A	M5A	0.78	2.54
			**0.73** (Av)	**1.39** (Av)
			0.05 (Sd)	0.93 (Sd)
M5	M2B	M3A	1.05	-0.17
	M3B	M4A	0.88	-0.84
			**0.97** (Av)	**-0.51** (Av)
			0.17 (Di)	0.67 (Di)
M6	M5A	M6B	0.84	-5.05
	M6A	M7A	0.86	-4.42
	M6C	M7D	0.83	-3.96
			**0.84** (Av)	**-4.48** (Av)
			0.02 (Sd)	0.55 (Sd)
M7	M6B	M7B	0.86	-1.81
	M7A	M8A	0.89	-1.68
	M7D	M8C	0.85	-2.11
	M8B	M9A	0.89	-2.27
			**0.87** (Av)	**-1.97** (Av)
			0.02 (Sd)	0.27 (Sd)
M8	M5A	M6C	0.71	4.76
	M6B	M7D	0.70	5.85
	M7A	M8B	0.70	4.31
	M7B	M8C	0.69	5.55
	M8A	M9A	0.70	3.72
	G1M7B	G1M8C	0.66	4.07
			**0.69** (Av)	**4.71** (Av)
			0.02 (Sd)	0.84 (Sd)

M9	M5A	M6A	0.89	-7.47
	M6B	M7A	0.91	-6.84
	M7B	M8A	0.94	-6.71
	M7D	M8B	0.91	-8.38
	M8C	M9A	0.95	-8.54
	G1M8C	G1M9A	0.91	-6.51
			**0.92** (Av)	**-7.41** (Av)
			0.02 (Sd)	0.88 (Sd)
Gc1	M7B	G1M7B	0.69	10.03
	M8C	G1M8C	0.66	8.55
	M9A	G1M9A	0.62	10.58
			**0.66** (Av)	**9.72** (Av)
			0.04 (Sd)	1.05 (Sd)
Gc2	G1M9A	G2M9A	**0.55**	**4.74**
Gc3	G2M9A	G3M9A	**1.00**	**-2.93**
F3	GN2	GN2F3	0.57	-8.99
	M1A	M1AF3	0.66	-7.69
			**0.62** (Av)	**-8.34** (Av)
			0.06 (Sd)	0.92 (Sd)
F3 with M3	M3BX	M3BF3X	**0.76**	**-11.66**
F6	GN	GNF6	0.68	9.43
	GN2	GN2F6	0.38	9.43
	M2A	M2AF6	0.37	9.08
	M3B	M3BF6	0.31	9.42
	M3BX	M3BF6X	0.14	9.64

	AG1	AG1F6	0.26	9.93
	AG2	AG2F6	0.29	8.19
	AG12	AG12F6	0.21	7.97
	AG12BS	AG12BSF6	0.24	9.50
	AG123	AG123F6	0.19	8.44
	MO1	MO1F6	0.23	8.53
	MO2	MO2F6	0.19	7.77
	BI	BIF6	0.23	7.69
	BI-G1	BIF6-G1	0.18	7.86
	BI-G2	BIF6-G2	0.21	7.82
	BIBS	BIBSF6	0.19	9.41
	TR123	TR123F6	0.19	7.87
	TR124	TR124F6	0.19	8.39
	TE	TEF6	0.19	7.87
	03S-BI	03S-BIF6	0.21	7.41
	30S-BI	30S-BIF6	0.21	7.43
	33S-BI	33S-BIF6	0.21	7.15
	06S-BI	06S-BIF6	0.24	8.22
	66S-BI	66S-BIF6	0.22	6.18
	36S-BI	36S-BIF6	0.21	7.70
	333S-TR124	333S-TR124F6	0.14	7.48
			**0.24** (Av)	**8.30** (Av)
			0.11 (Sd)	0.93 (Sd)
F3'	TR123	F3(3)TR123	0.75	-1.69
	TE	F3(3)TE	0.76	-1.59
			**0.76** (Av)	**-1.64** (Av)
			0.01 (Di)	0.10 (Di)
X	M1AF3	M1AF3X	**0.26**	**4.67**
X with M2	M3B	M3BX	0.49	-0.87

	M3BF6	M3BF6X	0.32	-0.65
	M4C	M4CX	0.44	-0.10
			**0.42** (Av)	**-0.54** (Av)
			0.09 (Sd)	0.40 (Sd)
BS	AG2F6	AG2BSF6	0.36	1.35
BS with GN1	AG12	AG12BS	0.29	14.25
	AG12F6	AG12BSF6	0.32	15.78
	AG123	AG123BS	0.18	19.62
	BI	BIBS	0.13	14.25
	BIF6	BIBSF6	0.09	15.97
	BIF6-G1	BIBSF6-G1	0.21	16.43
			**0.20** (Av)	**16.05** (Av)
			0.09 (Sd)	1.97 (Sd)
BS with GN1 & M4	GNM5A	GNM5ABS	**0.47**	**7.59**
GN1	M3B	AG1	0.39	-2.19
	M3BF6	AG1F6	0.34	-1.68
	AG2	AG12	0.34	-2.84
	AG4	AG14	0.30	-2.52
	AG2F6	AG12F6	0.26	-3.06
	AG2F3X	AG12F3X	0.21	-3.64
	AG24	AG124	0.26	-1.52
	M5A	GNM5A	0.26	-1.72
	MO2	BI-G1	0.27	-2.94
	MO2F6	BIF6-G1	0.26	-2.85
	3S-MO2F6	30S-BIF6-G1	0.21	-3.14
			**0.28** (Av)	**-2.55** (Av)
			0.06 (Sd)	0.69 (Sd)

GN1 with GN3	AG3	AG13	0.35	1.19
	AG23	AG123	0.30	0.08
	AG34	AG134	0.38	1.72
	AG234	AG1234	0.24	3.25
	M2AF6	AG1F6-M3	0.48	4.83
			**0.35** (Av)	**2.21** (Av)
			0.09 (Sd)	1.85 (Sd)
GN1 with GN3 & BS	AG2BSF6	AG12BSF6	**0.22**	**11.37**
GN2	M3B	AG2	0.40	10.62
	M3BF6	AG2F6	0.38	9.39
	M3BF3X	AG2F3X	0.33	6.89
	AG1	AG12	0.35	9.97
	AG3	AG23	0.38	9.92
	AG1F6	AG12F6	0.30	8.01
	AG13	AG123	0.33	8.01
	MO1	BI-G2	0.28	8.38
	MO1F6	BIF6-G2	0.26	7.67
	3S-MO1F6	03S-BIF6-G2	0.22	7.06
			**0.32** (Av)	**8.59** (Av)
			0.06 (Sd)	1.30 (Sd)
GN2 with GN4	AG4	AG24	0.42	-4.35
	AG14	AG124	0.37	-3.35
	AG34	AG234	0.39	-2.93
	AG134	AG1234	0.25	-1.40
			**0.36** (Av)	**-3.01** (Av)
			0.07 (Sd)	1.23 (Sd)
GN3	M3B	AG3	0.32	5.62
	AG2	AG23	0.30	4.92

	AG4	AG34	0.29	5.05
	AG24	AG234	0.26	6.47
			**0.29** (Av)	**5.52** (Av)
			0.03 (Sd)	0.71 (Sd)
GN3 with GN1	AG1	AG13	0.28	9.80
	AG12	AG123	0.26	7.84
	AG14	AG134	0.37	9.29
	AG12BS	AG123BS	0.15	13.21
	AG12F6	AG123F6	0.24	8.31
	AG124	AG1234	0.24	11.24
			**0.26** (Av)	**9.95** (Av)
			0.07 (Sd)	1.99 (Sd)
GN4	M3B	AG4	0.60	2.84
	AG1	AG14	0.51	2.51
	AG3	AG34	0.57	2.27
	AG13	AG134	0.60	2.00
			**0.57** (Av)	**2.41** (Av)
			0.04 (Sd)	0.36 (Sd)
GN4 with GN2	AG2	AG24	0.62	-12.13
	AG12	AG124	0.54	-13.65
	AG23	AG234	0.58	-10.58
	AG123	AG1234	0.52	-7.41
			**0.57** (Av)	**-10.94** (Av)
			0.04 (Sd)	2.67 (Sd)
G1	AG1	MO1	0.86	4.04
	AG1F6	MO1F6	0.83	2.64
	AG1F6-M3	MO1F6-M3	0.79	2.41
	BI-G1	BI	0.78	2.21

	BIF6-G1	BIF6	0.83	2.04
	BIBSF6-G1	BIBSF6	0.71	1.58
	30S-BIF6-G1	30S-BIF6	0.72	1.75
			**0.79** (Av)	**2.38** (Av)
			0.06 (Sd)	0.82 (Sd)
G2	AG2	MO2	0.80	1.91
	AG2F6	MO2F6	0.70	1.49
	BI-G2	BI	0.72	1.57
	BIF6-G2	BIF6	0.74	1.44
	03S-BIF6-G2	03S-BIF6	0.65	1.23
			**0.72** (Av)	**1.53** (Av)
			0.05 (Sd)	0.25 (Sd)
S13	BI	03S-BI	0.01	13.37
	30S-BI	33S-BI	0.09	12.94
	60S-BI	63S-BI	0.08	12.44
	BIF6-G2	03S-BIF6-G2	0.08	13.30
	BIF6	03S-BIF6	-0.01	13.09
	30S-BIF6	33S-BIF6	0.09	12.66
	MO1F6	3S-MO1F6	0.12	13.91
			**0.07** (Av)	**13.10** (Av)
			0.05 (Sd)	0.49 (Sd)
S16	BI	06S-BI	0.44	7.98
	60S-BI	66S-BI	0.49	7.04
	60S-BIF6	66S-BIF6	0.43	8.82
	BIF6	06S-BIF6	0.45	8.51
	30S-BIF6	36S-BIF6	0.51	7.89
	30S-BI	36S-BI	0.51	7.62
			**0.47** (Av)	**7.98** (Av)
			0.04 (Sd)	0.63 (Sd)

S23	BI	30S-BI	-0.03	12.58
	03S-BI	33S-BI	0.05	12.15
	06S-BI	36S-BI	0.04	12.35
	BIF6-G1	30S-BIF6-G1	0.06	12.61
	BIF6	30S-BIF6	-0.05	12.32
	03S-BIF6	33S-BIF6	0.05	11.89
	MO2F6	3S-MO2F6	0.11	12.90
			**0.03** (Av)	**12.40** (Av)
			0.05 (Sd)	0.33 (Sd)
S26	BI	60S-BI	0.40	11.71
	03S-BI	63S-BI	0.47	10.78
	06S-BI	66S-BI	0.45	10.77
	663S-TR123	063S-TR123	0.46	9.59
			**0.45** (Av)	**10.71** (Av)
			0.03 (Sd)	0.87 (Sd)

[1] A kind and position of each residue is indicated in Figure 2.
[2] Correspondence of the name and structure is listed in Table S1.
[3] Difference of *S* values on a size-fractionation HPLC between the PA-glycans with or without a residue concerned.
[4] Difference of *R* values on a reversed phase HPLC between the PA-glycans with or without a residue concerned.
[5] Partial elution times are indicated by bold letters.
[6] Average of the difference values for a residue concerned.
[7] Difference between the two difference-values for a residue concerned.
[8] Standard deviation of the three or more difference values for a residue concerned.
