# Supplementary material for: Improved Method for Drawing of a Glycan Map, and the First Page of Glycan Atlas, Which Is a Compilation of Glycan Maps for a Whole Organism
Source: PLoS One. 2014 Jul 9;9(7):e102219. doi: 10.1371/journal.pone.0102219 (PMC4090225; doi:10.1371/journal.pone.0102219)
Supplement: Table S3 — Mapping data of PA-N-glycans from human serum. (PDF) [file pone.0102219.s003.pdf]

Table S3. Mapping data of PA-*N*-glycans from human serum.

Symbol	*R* value	*S* value	Relative amounts	Corresponding *N*-glycans	*R* value[1]	*S* value[1]
O-1	30.8	2.5	6.6	03N-core1	30.5	2.5
O-2	41.5	3.6	1.9	36N-core1	40.0	3.5
N-1	32.7	8.4	0.5	M8A	32.7	8.4
N-2	36.4	9.1	0.6	M9A	36.4	9.1
N-3	41.4	6.6	1.4	M6B	41.2	6.6
N-4	46.1	5.8	2.2	M5A	46.2	5.8
N-5	51.3	6.5	0.4	M6C	51.0	6.5
N-6	52.4	4.9	0.4	AG12	52.3	4.9
N-7	54.3	5.6	0.4	BI-G1<br>BI-G2	54.1<br>54.8	5.6<br>5.7
N-8	56.4	6.4	0.7	BI	56.3	6.4
N-9	60.4	5.1	7.7	AG12F6	60.3	5.1
N-10	62.0	5.8	12.4	BIF6-G1	62.0	5.8

				BIF6-G2	62.6	5.9
N-11	63.8	6.5	7.2	BIF6	64.0	6.6
N-12	68.9	5.8	0.4	*BIBS-G1*[2]<br>*BIBS-G2*	*68.2*<br>*69.1*	*5.7*<br>*5.8*
N-13	76.1	5.4	1.6	AG12BSF6	76.1	5.4
N-14	78.3	6.0	2.3	BIBSF6-G1<br>*BIBSF6-G2*	78.4<br>*78.5*	6.0<br>*6.0*
N-15	79.9	6.7	0.9	BIBSF6	80.0	6.7
A1-1a	56.0	6.8	0.6	*6N-GalGNM4C*	*56.4*	*6.4*
A1-1b	56.0	7.7	0.5	*6N-GalGNM5A*	*55.9*	*7.4*
A1-2	63.4	7.1	23.4	06N-BI	64.3	6.8
A1-3	66.9	6.9	1.7	60N-BI	68.1	6.8
A1-4a	70.6	6.6	1.2	*06N-BIF-G2*	*70.6*	*6.4*
A1-4b	70.6	8.0	0.9	-[3]	-	-
A1-5	71.9	7.3	9.5	06N-BIF6	72.5	7.1
A1-6a	73.7	6.8	0.7	*60N-BIF6*	*74.7*	*7.1*

A1-6b	76.5	7.3	1.1	*06N-BIBS*	*78.6*	*7.0*
A1-7	86.2	7.6	3.6	*06N-BIBSF6*	*88.0*	*7.2*
A2-1	69.0	7.7	1.2	-	-	-
A2-2	70.5	8.9	0.9	-	-	-
A2-3	74.6	7.4	<u>100</u>[4]	66N-BI	75.2	7.3
A2-4	76.1	7.0	5.7	36N-BI	76.5	6.9
A2-5a	79.7	8.0	1.8	063N-TR123	79.8	7.7
A2-5b	79.7	9.1	0.9	-	-	-
A2-6a	81.1	7.7	4.8	66N-BIF6	81.4	7.5
A2-6b	81.1	8.4	2.5	-	-	-
A2-7a	84.4	7.3	0.5	36N-BIF6	84.2	7.1
A2-7b	84.4	8.5	1.1	-	-	-
A2-8	94.5	7.6	2.8	*66N-BIBSF6*	*97.5*	*7.7*
A3-1	87.5	8.8	4.3	-	-	-
A3-2	89.9	8.2	10.6	663N-TR123	89.4	8.2

A3-3	94.3	8.7	3.7	666N-TR123	93.7	8.6
A4-1	88.7	9.1	0.9	-	-	-

[1] The values of standard PA-glycans were from Table S1.
[2] Data written by italic letters were estimated values by using of the partial elution times on an additivity rule.
[3] No corresponding PA-glycan was found.
[4] The amount of this PA-glycan species was taken as 100.
