# Supplementary material for: Improved Method for Drawing of a Glycan Map, and the First Page of Glycan Atlas, Which Is a Compilation of Glycan Maps for a Whole Organism
Source: PLoS One. 2014 Jul 9;9(7):e102219. doi: 10.1371/journal.pone.0102219 (PMC4090225; doi:10.1371/journal.pone.0102219)
Supplement: Table S4 — Proposed structures of human serum glycans. (PDF) [file pone.0102219.s004.pdf]

Table S4. Proposed structures of human serum glycans.

Symbol	Structure[1]	Abbreviation
O-1	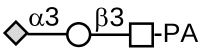	03N-core1
O-2	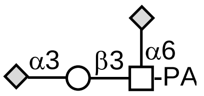	36N-core1
N-1	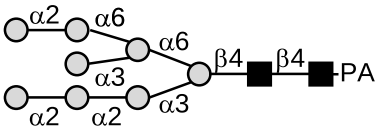	M8A
N-2	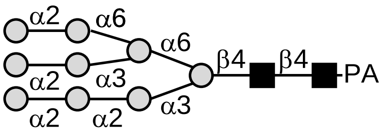	M9A
N-3	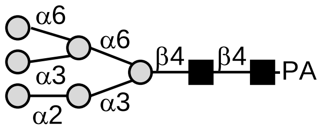	M6B
N-4	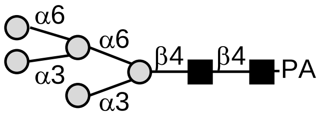	M5A
N-5	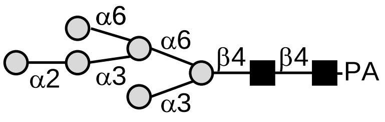	M6C
N-6	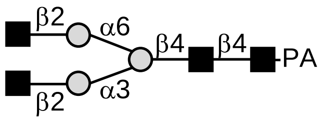	AG12
N-7	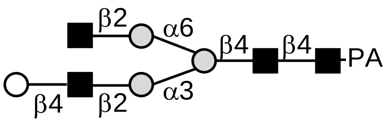	BI-G2
N-8	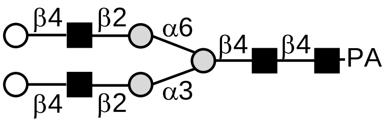	BI

N-9

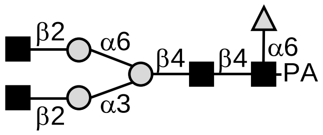

AG12F6

N-10

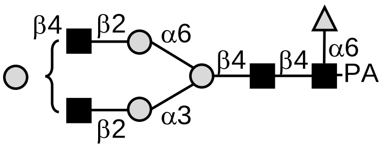

BIF6-G

N-11

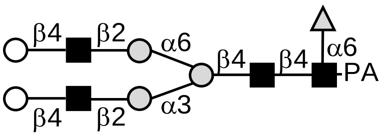

BIF6

N-12

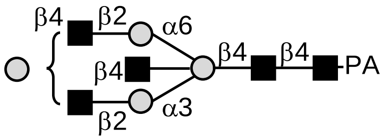

BIBS-G

N-13

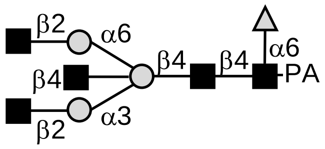

AG12BSF6

N-14

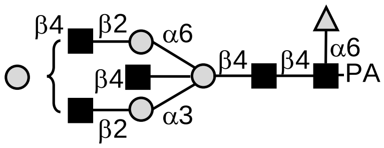

BIBSF6-G

N-15

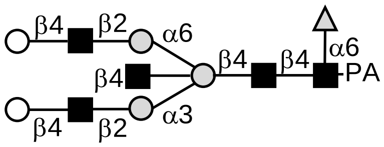

BIBSF6

A1-1a

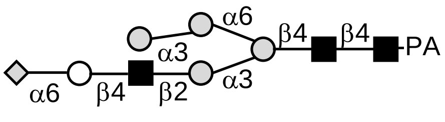

6N-GalGNM4C

A1-1b

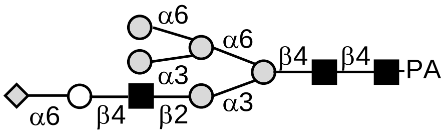

6N-GalGNM5A

A1-2

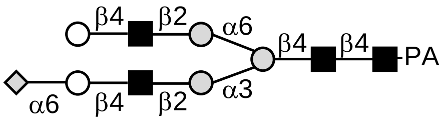

06N-BI

A1-3	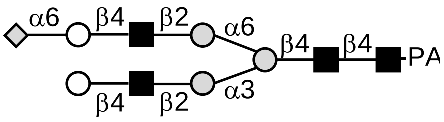	60N-BI
A1-4a	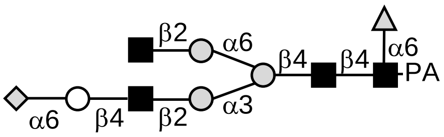	06N-BIF-G2
A1-4b	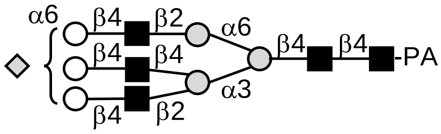	6N-TR123
A1-5	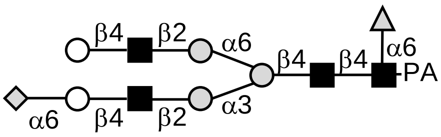	06N-BIF6
A1-6a	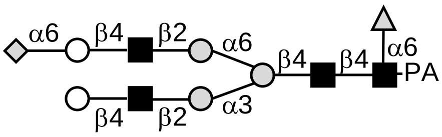	60N-BIF6
A1-6b	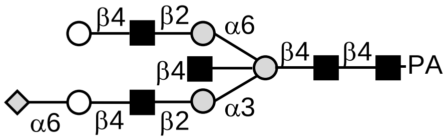	06N-BIBS
A1-7	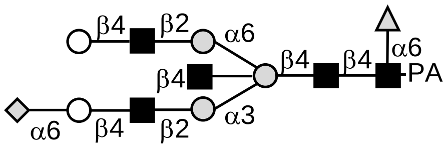	06N-BIBSF6
A2-2	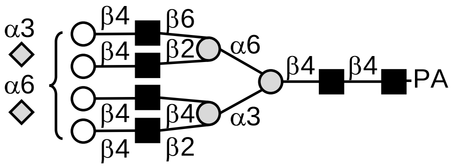	(36)N-TE
A2-3	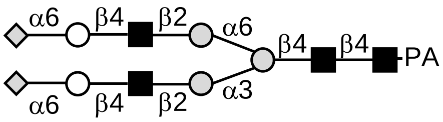	66N-BI
A2-4	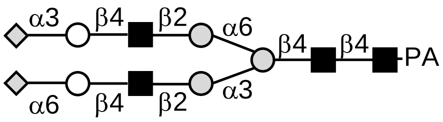	36N-BI

A2-5a

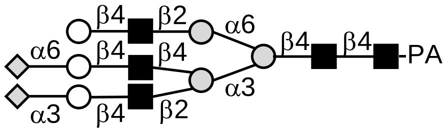

063N-TR123

A2-5b

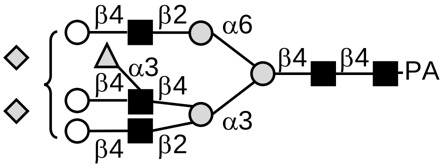

diN-F3(3)TR123

A2-6a

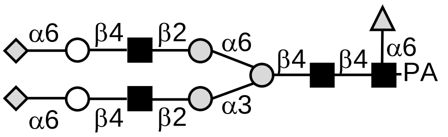

66N-BIF6

A2-6b

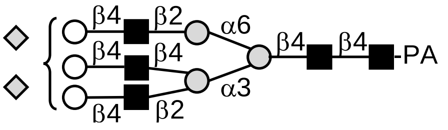

diN-TR123

A2-7a

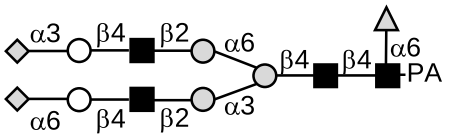

36N-BIF6

A2-7b

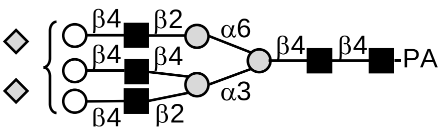

diN-TR123

A2-8

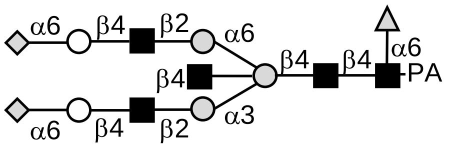

66N-BIBSF6

A3-1

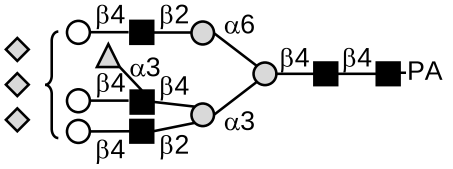

triN-F3(3)TR123

A3-2

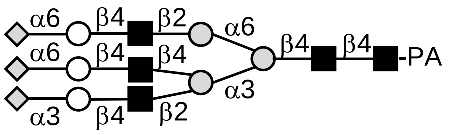

663N-TR123

A3-3

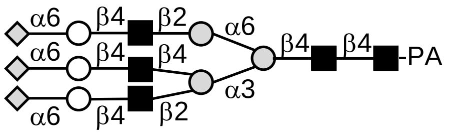

666N-TR123

A4-1	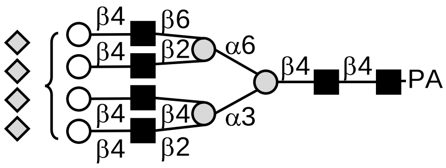	tetN-TE

[1] Symbols of monosaccharides are the same as in Table S1.
