# Supplementary material for: Improved Method for Drawing of a Glycan Map, and the First Page of Glycan Atlas, Which Is a Compilation of Glycan Maps for a Whole Organism
Source: PLoS One. 2014 Jul 9;9(7):e102219. doi: 10.1371/journal.pone.0102219 (PMC4090225; doi:10.1371/journal.pone.0102219)
Supplement: Table S5 — Sialidase digestions of PA-N-glycans from human serum. (PDF) [file pone.0102219.s005.pdf]

Table S5. Sialidase digestions of PA-*N*-glycans from human serum

Symbol	Shifted position after *Arthrobactor* sialidase digestion	Liberated sialic acid by α2-3sialidase digestion
A1-1a	GalGNM4C	0
A1-1b	GalGNM5A	0
A1-2	BI	0
A1-3	BI	0
A1-4a	BIF6-G2	0
A1-4b	TR123	0
A1-5	BIF6	0
A1-6a	BIF6	0
A1-6b	BIBS	0
A1-7	BIBSF6	0
A2-1	(no corresponding standard)	1
A2-2	TE	1
A2-3	BI	0
A2-4	BI	1
A2-5a	TR123	1
A2-5b	F3(3)TR123	ND[1]
A2-6a	BIF6	0
A2-6b	TR123	ND
A2-7a	BIF6	1
A2-7b	TR123	ND
A2-8	BIBSF6	0

A3-1	F3(3)TR123	ND
A3-2	TR123	1
A3-3	TR123	0
A4-1	TE	ND

[1] Not determined.
